# Genetic Analysis of Platelet-Related Genes in Hepatocellular Carcinoma Reveals a Novel Prognostic Signature and Determines PRKCD as the Potential Molecular Bridge

**DOI:** 10.1186/s12575-022-00185-9

**Published:** 2022-12-03

**Authors:** Xiangyu Li, Kai Zhao, Yun Lu, Jianming Wang, Wei Yao

**Affiliations:** 1 Department of Biliary and Pancreatic Surgery/Cancer Research Center Affiliated Tongji Hospital, Tongji Medical College, Huazhong University of Science and Technology, Wuhan, 430030 Hubei China; 2 Affiliated Tianyou Hospital, Wuhan University of Science & Technology, Wuhan, 430064 China; 3 Department of Oncology Affiliated Tongji Hospital, Tongji Medical College, Huazhong University of Science and Technology, Wuhan, 430030 Hubei China

**Keywords:** Platelet, Hepatocellular carcinoma, PRKCD, Cross-talk, Prognosis, Immunity

## Abstract

**Background:**

Hepatocellular carcinoma (HCC) belongs to a representative lethality gastrointestinal malignancy, and comprehensive management of HCC remains intractable at present on account of its invasive biological feature that is easy to relapse and early metastasis. The intimate connection between platelets and tumor progression has been widely reported, and platelet-related indicators are also used in the clinical practice of carcinoma. This work is designed to investigate the significance of platelet-related genes in the prognostic prediction of patients with HCC and their potential role in the cross-talk between HCC cells and platelets in the tumor microenvironment.

**Methods:**

By integrating the RNA-seq data and clinicopathological information of HCC patients, we extracted prognosis-associated platelet-related genes based on the univariate cox analysis and further established a relevant prognostic signature via the lasso cox regression analysis, and two independent HCC cohorts were used as external validation. Multiple bioinformatics methods were utilized to explore the underlying functional discrepancy between different risk groups classified by the risk model. And in vitro proliferation, invasion, and migration assays were conducted to investigate the effect of platelet stimulation on HCC cells’ viability and motility, and flow cytometric analysis was exerted to demonstrate the influence of HCC cells on platelet activation.

**Results:**

A novel platelet-related risk model was developed and patients both in the training and testing cohorts were divided into distinct risk subgroups according to the median risk score. It was observed that the high-risk status was closely associated with poor prognosis and worse clinicopathological parameters. Meanwhile, an obvious discrepancy in the constitution of the immune microenvironment also indicated that distinct immune status might be a potential determinant affecting prognosis as well as immunotherapy reactiveness. Moreover, in vitro experiments demonstrated that PRKCD could act as a molecular bridge between tumor cells and platelets, which could either participate in regulating tumor malignant phenotype or mediating platelet activation.

**Conclusions:**

In brief, this work reveals a novel platelet-related risk signature for prognostic evaluation of HCC patients and confirms that PRKCD is a key messenger in HCC cell-platelet interaction and plays a crucial role in mediating platelet-induced tumor progression.

**Supplementary Information:**

The online version contains supplementary material available at 10.1186/s12575-022-00185-9.

## Introduction

HCC, the major type of primary liver cancer, remains a serious challenge for global public health management at present. Cancer statistics indicate that HCC ranks in the top 10 among all malignant tumors in both morbidity and mortality [1]. In China and most areas around the world, the burden of HCC in males is significantly higher than that in females, which may be attributed to relevant risk factors, including chronic viral hepatitis, excessive drinking, nonalcoholic fatty liver disease, as well as exposure to aflatoxin and other alimentary toxins [2, 3]. Although radical surgery and minimally invasive treatment have significantly improved the prognosis of patients with early HCC, most individuals are diagnosed already at later stages that are not suitable for surgery, and due to regional differences in prevention awareness and medical resources, the overall prognosis of HCC patients varies markedly [3]. Therefore, combined with the current status of HCC, further strengthening its early diagnosis and management is of great significance to improve its long-term prognosis.

Abnormal changes in platelets can mediate tumor malignant progression and chemotherapy-resistant through a diverse range of mechanisms, which is a longstanding and widely recognized concept [4]. Generally speaking, the aberrant increase of platelet counts is considered to assist in the early diagnosis of multiple occult malignancies [5], which can also be utilized as a strong predictor of survival in patients receiving corresponding treatments [6, 7]. Theoretically, platelets promote aggressive tumor growth through direct contact or paracrine pathways, at the same time, tumor cells can also stimulate platelets activation to further magnify this process, thus it can be seen that the cross-talk between cancer cells and platelets plays an essential part in the regulation of tumor malignant phenotype, which also provides more perspectives for the exploitation of cancer therapeutic targets by aiming at this kind of interaction [8]. The liver is a substantial organ with a dual blood supply system, and this anatomical characteristic also makes it necessary to explore the potential function of platelets in liver diseases, especially in HCC. In current studies, clinical explorations about platelets in HCC mainly concentrate on the significance of the variation of platelet counts or morphology as well as the platelet-related ratio in prognostic prediction [9–11]. While from the perspective of clinical findings combined with molecular changes, whether there are corresponding abnormal changes in platelet-related genes in the development of HCC remains unclear.

In the current research, for an in-depth understanding of the value of platelet in the prediction and prognostic evaluation of HCC, we collected and arranged platelet-related genes and then established a risk signature via the least absolute shrinkage and selection operator (LASSO) cox regression analysis, and the validation of the predictive performance was accomplished via multiple independent datasets, meanwhile, the differences in biological function and immune status of HCC patients at high or low risk were also investigated and described briefly. Subsequently, we illustrated the pivotal role of the representative risk gene we selected in mediating platelet-induced tumor malignant biological behaviors and its potential messenger function in the platelet activation process stimulated by cancer cells through in vitro experiments. The results of this work have strengthened the recognition of the cross-talk between HCC cells and platelets from a molecular perspective, which will be conducive to the prognostic appraisement of HCC patients and help the exploration of novel therapeutic targets.

## Materials and Methods

### Data Acquisition and Handling

The open-end HCC RNA-seq datasets and matching available clinicopathology information were acquired from The Cancer Genome Atlas Dataset (TCGA) (https://portal.gdc.cancer.gov/), Gene Expression Omnibus (GEO) Dataset (http://www.ncbi.nlm.nih.gov/geo/), and the International Cancer Genome Consortium (ICGC) portal (https://dcc.icgc.org/). 370 HCC cases in the TCGA-LIHC cohort with complete survival and pathological data were extracted as a training set. And the Ensemble IDs in the raw expression matrix were transformed into corresponding gene symbols using Perl language, then the initial gene expression profile (FPKM data) was converted into the TPM data for further informatics analysis. Additionally, 221 HCC cases in the GSE14520 cohort and 227 cases in the ICGC-LIRI cohort with available expression data and clinical information were acquired as external independent validation datasets. RNA-seq information for the three cohorts and the corresponding survival and pathology data are presented in Supplementary files 1, 2, 3, 4, 5, 6. Furthermore, relevant genes involved in platelet activation, aggregation, and signal transduction were collected and collated from the MSigDB database (http://www.broad.mit.edu/gsea/msigdb/). All bioinformatic analyses in this work were conducted with the R software (version 4.0.4).

### Recognition of Platelet-Related Genes

300 platelet-related genes (PRGs) in total associated with platelet biological functions were extracted and listed in Table S1 (Supplementary table), and PRGs with differential expression (DEPRGs) between normal and tumor samples in the training set were subsequently filtrated out that satisfied the thresholds of |log_2_FC| > 1 and *p* < 0.01 based on the “limma” package (version 3.46.0). The protein interaction network among these DEPRGs was exhibited using the STRING online platform (https://string-db.org/). Furthermore, both Gene Ontology (GO) and Kyoto Encyclopedia of Genes and Genomes (KEGG) analyses were employed to reveal the functions or processes of these DEPRGs enriched.

### Identification of Prognostic PRGs

DEPRGs that conform to the screening criteria were acquired for the uni-cox analysis (*p* < 0.01) to notarize the prognosis-associated PRGs, and their correlation network was displayed based on the R “igraph” (version 1.3.0), “psych” (2.2.5) and “reshape2” (1.4.4) packages. Kaplan-Meier (KM) survival curves of each PRG were portrayed to exhibit the prognostic performance according to their expression levels.

### Subtype Clustering Analysis

Unsupervised consensus cluster analysis was conducted to divide HCC patients in the training set into different subtypes according to the prognostic PRGs via the R “ConsensusClusterPlus” package (version 1.54.0). Overall survival (OS), as well as progression-free survival (PFS) curves, were visualized utilizing the R “survminer” package (0.4.9). By integrating corresponding clinicopathological information (including TNM stage, tumor stage, pathological grade, patient’s gender, and age), the discrepancies in clinical characteristics between different subtypes were displayed with heatmap via the R “pheatmap” package (1.0.12). Additionally, Gene Set Variation Analysis (GSVA) was applied to investigate the functional enrichment pathways between distinct subtypes according to the reference KEGG data set (c2.cp.kegg.v7.4.symbols.gmt). Single sample gene set enrichment analysis (ssGSEA) was exerted to excavate the differences in tumor-infiltrating immunocytes and immunological functions between HCC subtypes via the R “GSVA” package (1.38.2).

### Establishment and Verification of Platelet-Associated Risk Signature

Based on the prognostic PRGs extracted by uni-cox analysis previously, lasso-cox regression analysis was further performed to obtain representative genes to establish prognostic risk signature, and with the assistance of the R “glmnet” package (version 4.1–4), a 12 PRGs involved risk model was ultimately constructed, and HCC patients in the TCGA-LIHC cohort were classified into two distinct risk subgroups based on the median risk score which was calculated via the following formula: Risk Score = α_i_ × β_i_ PRGs (α_i_ represents the regression coefficient of each PRG, β_i_ represents the expression level of each PRG). KM analysis was employed to describe and compare the survival curves between two risk groups. The risk scoring distribution, survival condition, as well as risk gene expression pattern between the two groups were also displayed through the R “pheatmap” package. Principal Component Analysis (PCA), which refers to a widely used data dimension reduction algorithm, was carried out to visualize the differences between distinct subgroups. The receiver operating characteristic (ROC) curve and the area under the curve (AUC) were employed to evaluate the accuracy of the risk signature via the R “timeROC” package (version 0.4). Additionally, aiming to improve the stability and reliability of the model, we further performed external verification both in the GSE14520 and ICGC-LIRI cohorts.

### Correlation between Risk Signature and Clinicopathological Parameters

To reveal the latent clinical significance of the risk model, the correlation between risk score and clinicopathological features was assessed via the R “ComplexHeatmap” package (version 2.6.2), and using uni- and multivariate (multi-) cox analyses to determine the independent prognostic value of risk scores both in the training and validation cohorts.

### Subgroup Prognostic Evaluation

HCC cases in the TCGA-LIHC cohort were stratified into different clinical subgroups as follows: age (> 65 and < = 65), gender (female and male), TNM stage (T1–2, 3–4, N0–1, and M0–1), tumor stage (stage I-II, III-IV), and pathological grade (G1–2, 3–4). KM survival curves were further conducted to assess the diagnostic capability of the prognostic signature in two risk groups with different clinical parameters.

### Functional Enrichment Analysis of the Signature

The discrepancy in biological process (BP), molecular function (MF), and cellular components (CC), as well as functional pathways between two risk groups, was investigated through GSVA and GSEA methods by using the “clusterProfiler” package in R (version 3.18.1). Multiple immune infiltration algorithms as well as ssGSEA method were performed to investigate the differences in tumor-infiltrating immunocytes and immunological functions between two distinct risk groups.

### Genetic Mutation and Drug Susceptibility Analysis

The somatic mutation information was downloaded and collated from the online TCGA database, and the mutation landscape of HCC patients at different risk statuses was visualized with waterfall plots via the “maftools” package in R (version 2.6.05). The prediction of immunotherapeutic responsiveness of HCC samples was based on the tumor immune dysfunction and exclusion (TIDE) algorithm. Estimation of sensitivity of common chemotherapeutic agents in patients with HCC was conducted via the “pRRophetic” package (version 0.5) according to the online Genomics of Drug Sensitivity database (https://www.cancerrxgene.org/), and the results were calculated and represented with the half-maximal inhibitory concentration (IC_50_).

### Cell Lines and Cultivation Condition

Two human HCC cell lines, comprising HepG2, and MHCC97H, were purchased from the Center for Type Culture Collection (CTCC, China), which had been examined for short tandem repeat (STR) and excluded from mycoplasma infection. Both cell lines were cultivated in 10% fetal bovine serum (FBS) (Gibco, USA) replenished Dulbecco’s modified Eagle medium (DMEM) (Gibco, USA) and incubated at 37% filled with 5% CO_2_.

### Cell Transfection

With the purpose of down-regulating the expression of PRKCD in HCC cells, siRNAs were selected for targeted gene silencing and their primer sequences were listed below: negative control siRNA (si-NC): sense: 5′-UUCUCCGAACGUGUCACGUTT-3′, anti-sense: 5′-ACGUGACACGUUCGGAGAATT-3′; si-PRKCD-1: sense: 5′-CCAUGGUGAUGAUGAGGAUTT-3′, anti-sense: 5′-AUCCUCAUCAUCACCAUGGTT-3′; si-PRKCD-2: sense: 5′-GCAGCAAGUGCAACAUCAATT-3′, anti-sense: 5′-UUGAUGUUGCACUUGCUGCTT-3′), which were synthesized and obtained from the DesignGene Biotechnology (Shanghai, China). HCC cells in well condition were uniformly seeded into 6-well plates in advance, and transfection was performed with Lipofectamine 2000 (Invitrogen, CA, USA) following the manufacturer’s instruction when the cells were adherent and reached 70% confluence.

### Immunohistochemistry Analysis

Immunohistochemical (IHC) staining was exerted to analyze the relative expression of proteins between normal and tumor tissue specimens, and the results were acquired from the Human Protein Atlas (HPA) database (https://www.proteinatlas.org/).

### Real-Time Quantitative PCR (RT-qPCR)

Total cellular RNA was distilled utilizing TRIzol reagent (Vazyme, Nanjing, China) according to the operating instruction. ABScript III RT Master Mix (ABclonal, Wuhan, China) was used for the synthesis of the cDNA template, which was subsequently applied for quantitative PCR assay via the Universal SYBR Green Fast qPCR Mix (ABclonal) according to the operating scheme in the detection system (Bio-Rad, CA, USA). Beta-Actin (ACTB) was optioned as negative control and the relative mRNA expression of target genes were measured via the 2^-∆∆Ct^ algorithm. All primer sequences used in this work were synthesized by Sangon Biotech (Shanghai, China) and listed in Table S2 (Supplementary table).

### Western Blotting

The treated HCC cells were lysed with RIPA buffer added both protease and phosphatase inhibitors, and then transferred to a centrifuge tube and centrifuged for 15 min at 4 °C, 12000 rpm, the lysate supernatant was further gathered and boiled with 5 × loading buffer for 10 min at 95 °C. Subsequently, the extracts were separated on sodium dodecyl sulfate polyacrylamide (SDS-PAGE) gels, transferred to 0.45 μm polyvinylidene fluoride (PVDF) membranes, sealed at room temperature with 5% skim milk, then incubated with primary antibody (anti-PRKCD, 1:1000, a17427, ABclonal) for 16 hours at 4 °C, subsequently incubated with appropriate secondary antibodies for 2 hours at normal temperature. Finally, protein bands were visualized via the chemiluminescence imaging system, and the results were analyzed in Image Lab software (Bio-Rad). For the comparison among different treatment groups, the GAPDH mouse monoclonal antibody (1:20000, ac033, ABclonal) was used as an internal negative control.

### In Vitro Proliferation, Invasion, and Migration Assays

Both cell counting kit-8 (CCK-8, ABclonal) assay and colony formation test were employed to determine the viability of HCC cells in vitro. As for the CCK-8 assay, HepG2 and MHCC97H cells received different treatments were cultivated into a 96-well plate with approximately 3000 cells per well, after the cells were adherent, replaced the original medium with 100 μl DMEM supplemented with 10 μl CCK-8 reagent and co-incubated for 2 hours at 37 °C, followed by measuring cellular absorbance at 450 nm. Six repeated measurements were performed in each time period of all experimental groups, and the whole test lasted for 3 days. For the colony formation test, the treated cells were cultivated into a 6-well plate with about 500 cells per well and cultured in the complete medium that refreshed every 72 hours and incubated as previously described. After 14 days of growth, the cell colonies were firstly immobilized with paraformaldehyde (Solarbio Science & Technology Co., Beijing, China) for 15 min, and then dyed with 1% crystal violet solution (G1014, Servicebio, Wuhan, China) for another 20 min. Finally, the colony formation numbers (more than 50 cells each colony) were enumerated using a microscope.

Both transwell assay and wound-healing test were exerted to assess the migration capacity of HCC cells. With regard to the transwell assay, tumor cells were first starved in serum-free DMEM for 8 h, then 5 × 10^4^ cells in 200 μl serum-free DMEM were seeded into the upper transwell chambers (Corning, USA) paved with or without Matrigel (BD Bioscience, USA) (respectively for invasion and migration), and 700 μl complete medium was added to the lower wells, followed by incubation for an appropriate time at 37, 5% CO_2_ atmosphere. Subsequently, the cells on the upper surface of the chambers were gently wiped and those on the lower surface were immobilized and dyed as we mentioned before, then the average migrated or invaded cells of each treated group were counted with ImageJ software from 5 randomly selected visual fields. As for the wound-healing test, differently processed tumor cells were planted into a 6-well plate and scratched with the 200 μl pipette tip when the cells reached more than 95% confluence, which was then washed and photographed at 0, 12, 24, and 48 h, finally, the migration ratio was assessed using ImageJ software. All the above functional experiments were performed in three independent replicates.

### Flow Cytometric Analysis

The negative control as well as PRKCD-silencing HCC cells were first seeded into a 24-well plate at 80% density. After cell adhesion, platelets were added and incubated in direct contact with tumor cells for 30 min and then collected for flow cytometry. The platelet surface markers P-selectin (CD62P) and CD41/CD61 (PAC-1) were used to assess the level of platelet activation, and the antibody CD61 was used specifically to determine the platelets. The platelet activation was detected by Beckman Coulter CytoFlex flow cytometry. All experiments were repeated independently at least three times. The main antibodies are shown: FITC anti-human CD61 (336,404; Biolegend, USA), PE anti-human CD62P (304,906; Biolegend, USA) and Alexa Fluor® 647 anti-human CD41/CD61 (362,806; Biolegend, USA). And the results were analyzed with the FlowJo software.

### In Vitro co-Culture System

Human peripheral blood was pulled out from an elbow vein into a silicified vacuum blood collection tube comprising 1:9 (v/v) 3.8% sodium citrate. The needed platelet-rich plasma (PRP) was gathered by centrifugation of whole blood for 15 min at 150 x g, which was then resuspended and washed using Tyrode buffer (components including 137 mM NaCl, 13.8 mM NaHCO3, 5.5 mM glucose, 2.5 mM KCl, 20 mM HEPES, as well as 0.36 mM-NaH2PO4; pH 7.4) for 15 min at 800 x g to harvest platelets. Suspended in Tyrode buffer without PGE1 or EDTA. All the above platelet preparations were carried out at normal temperature. Platelets were activated using 20 mM ADP and co-cultured with tumor cells, adjusting the ratio of tumor cells to platelets to 1:100.

### Statistical Analysis

Bioinformatic analysis and visualization in our research were achieved by the R software and its attached packages, and the results of in vitro experiments were representative images from three times independent repeats and presented by Means ± standard deviation (SD) in GraphPad Prism 9.0 software. Survival comparison was accomplished through the log-rank test and visualized with the KM curves. Employing Chi-square test to make a comparison between categorical variables. Employing Student’s t-test to assess the individual differences between distinct groups. Both uni- and multi-cox regression analyses were exerted to determine the independent prognostic efficacy of the risk score in multiple HCC cohorts. Using Spearman’s correlation test to measure the latent correlation between risk genes and immunocytes. One-way ANOVA was conducted to analyze the in vitro proliferation and migration assays. For each statistical analysis, *p* < 0.05 was regarded as the critical value of significant difference.

## Results

### Research Schematic Diagram

**Fig. 1:**
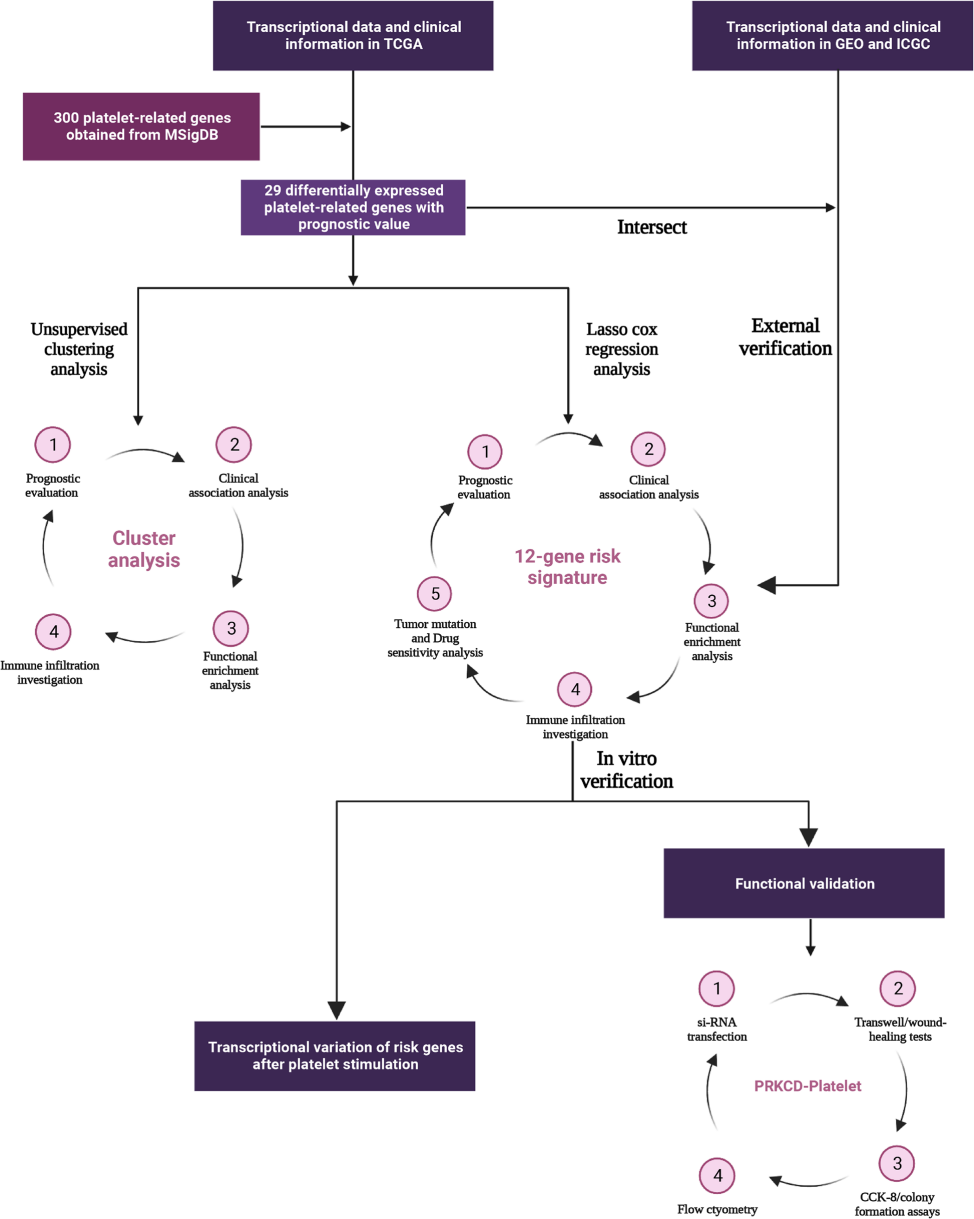
Schematic diagram of this work

Figure 1 displayed the overall flow sketch map of this work.

### Differential Expression Definition and Functional Annotation of PRGs

**Fig. 2:**
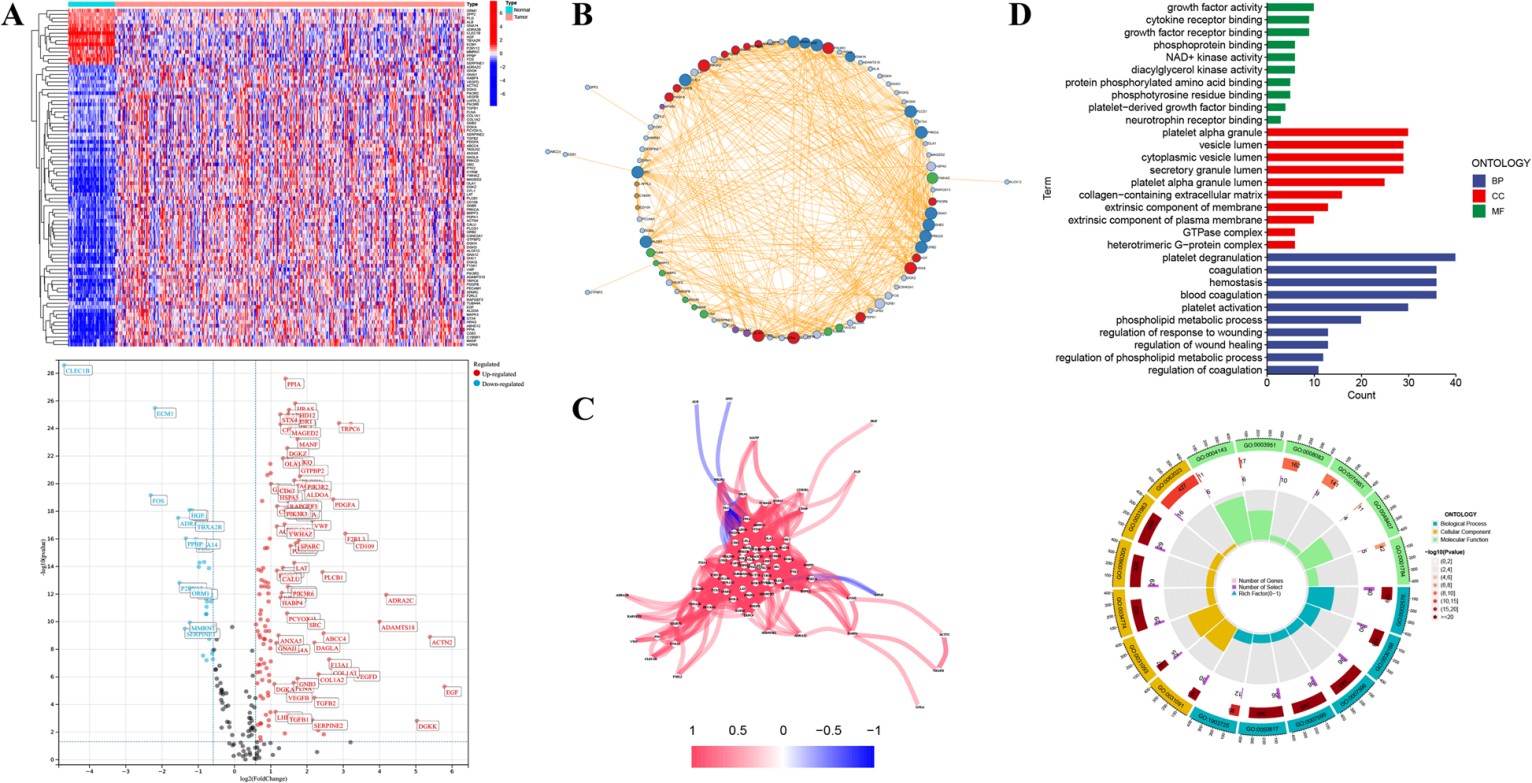
Expression and functional annotation of platelet-related genes (PRGs). **A** PRGs with differential expression (DEPRGs) in the TCGA-LIHC cohort that satisfied the screening threshold (|log_2_FC| > 1, *p* < 0.01). **B** Protein-Protein interaction (PPI) network of DEPRGs. **C** Regulatory relationship map of DEPRGs. **D** Functional annotation of these DEPRGs based on GO enrichment analysis

DEPRGs in the TCGA-LIHC cohort were extracted complying with the filtering criterion (|log_2_FC| > 1, *p* < 0.01), the results indicated that 90 PRGs were up or down-regulated in tumor tissues compared to the normal specimens in the training set. More specifically, 15 PRGs were up-regulated in normal specimens, while the remaining 75 PRGs were raised in HCC tissues relatively (Fig. 2A). PPI analysis with high confidence (0.7) was performed to describe the functional association network of these DEPRGs (Fig. 2B). Additionally, the correlation coefficients among DEPRGs were calculated that satisfied the correlation threshold (cutoff > 0.3), and as Fig. 2C exhibited, most PRGs possessed a positive regulatory relationship, except for a negative association among PLG, ORM1, SPP2, HRAS, PPIA, and ALB. Subsequently, to make a better recognition of the potential biological functions of these DEPRGs, GO enrichment analysis was exerted and the results implied that the GO terms observably enriched by PRGs were “platelet degranulation and activation” in BP, “platelet alpha granule” in CC, as well as “growth factor activity” in MF, respectively, implying that these differential genes were closely related to platelets (Fig. 2D).

### Identification of Prognostic PRGs

**Fig. 3:**
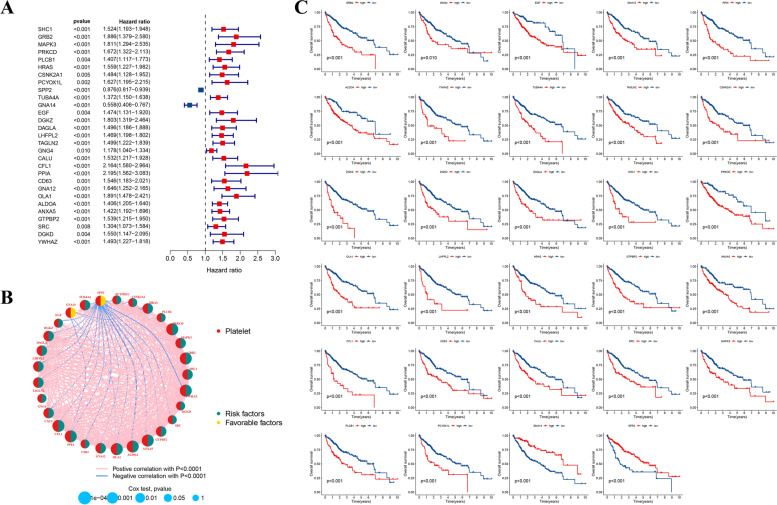
Recognition of prognosis-related PRGs. **A** 29 prognostic PRGs with respective hazard ratios were displayed with the forest plot via univariate cox analysis (*p* < 0.01). **B** Interaction network of prognostic PRGs. **C** The overall survival (OS) curves of HCC cases distinguished by the expression of these prognostic PRGs

Based on 90 DEPRGs, we further conducted the uni-cox regression analysis (*p* < 0.01) to obtain prognosis-associated PRGs. As the forest plot displayed in Fig. 3A, 29 PRGs with prognostic values were identified, and except for SPP2 (HR: 0.876, 95%CI: 0.817–0.939) and GNA14 (HR: 0.558, 95%CI: 0.406–0.767), the remaining PRGs were regarded as risk factors for HCC patients in the training set. And their correlation network was also displayed in Fig. 3B. Furthermore, the influence of prognostic gene expression on OS of HCC patients was displayed with KM survival curves more intuitively (Fig. 3C).

### Clustering Analysis of HCC Subtypes Based on 29 Prognosis-Related PRGs

**Fig. 4:**
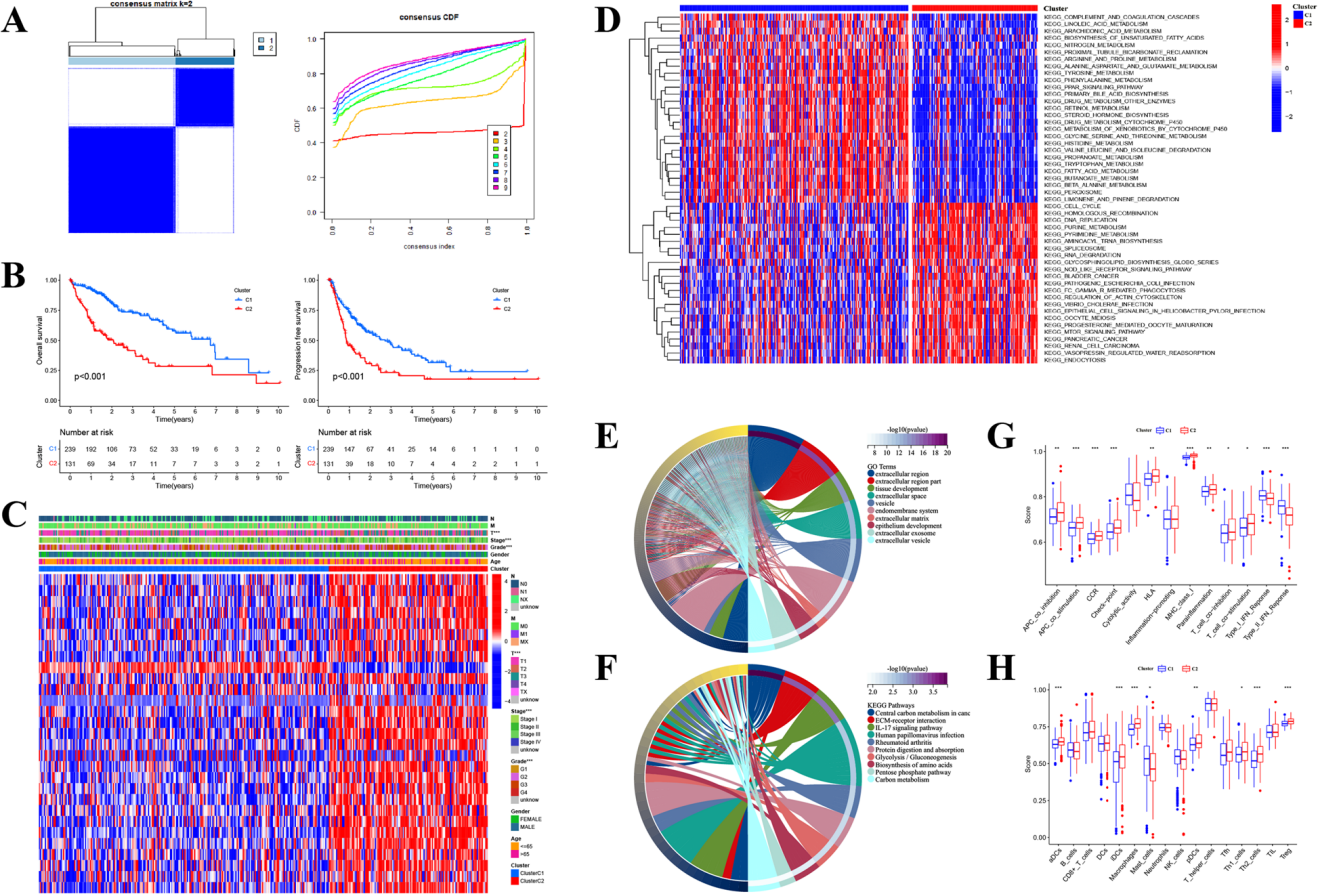
Clustering analysis of prognostic PRGs in the TCGA-LIHC cohort. **A** The whole patients were classified into two discrepant clusters (k = 2) according to the unsupervised clustering analysis. **B** The OS as well as progression-free survival (PFS) curves of two distinct clusters. **C** Heatmap of connection between clinicopathological parameters and HCC clusters. **D** Gene set variation analysis (GSVA) disclosed the respective enriched functional accesses in two different clusters. **E** The chordal plot of the GO terms and (**F**) the KEGG pathways enriched by differentially expressed genes (|log_2_FC| > 2, *p* < 0.01) between two distinct clusters. **G** The discrepancies in the composition of infiltrating immunocytes between distinct clusters. **H** The boxplot displayed the discrepancies in immune-related functions between distinct clusters. (*p < 0.05; **p < 0.01; ***p < 0.001)

Based on 29 prognostic PRGs, we further utilized the unsupervised clustering analysis to recognize different HCC subtypes. And two distinct clusters (cluster C1: 239 cases, cluster C2: 131 cases) were determined in the TCGA-LIHC cohort (Fig. 4A). Both OS and PFS time demonstrated that there was an obvious survival discrepancy between the two HCC subtypes, a poorer prognosis was observed in patients belonging to cluster C2 compared with those in cluster C1 (Fig. 4B). Whereafter, the intrinsic connection between HCC clusters and clinicopathological parameters was analyzed, and Fig. 4C displayed that cases in cluster C2 were closely associated with the higher expression of most PRGs and worse clinical features including T stage, tumor stage, and pathological grade, which also confirmed the poor prognosis of this HCC subtype. To further explore the discrepancies in functional pathways and immune features between different clusters, we performed GSVA and ssGSEA analyses in R software. As Fig. 4D displayed, compared to cluster C1, the C2 subtype mainly enriched in KEGG pathways like Cell cycle, Homologous recombination, DNA replication, cancer-associated (like Bladder, Pancreatic, and Renal cell carcinomas) pathways, and signaling transduction axis including NOD-like receptor and mTOR signaling pathways, implying that cluster C2 might own a close association with the occurrence and evolution of HCC. Furthermore, employing GO and KEGG analyses to make a functional annotation of the DEGs identified between two different subtypes (|log_2_FC| > 2, *p* < 0.01) and the top 10 enriched terms of both were displayed respectively (Fig. 4E-F). Moreover, according to the immune infiltrating scores calculated by the ssGSEA algorithm, several immunocytes (including aDCs, iDCs, pDCs, Macrophages, Th1 and Th2 cells, as well as Tregs) were observed that had a higher infiltrating level in cluster C2, while Mast cells had a higher infiltrating score in C1 subtype (Fig. 4G). Similarly, most immunological functions like APC co-inhibition and co-stimulation, CCR, Check-point, HLA, MHC class I, Para-inflammation, as well as T cell co-inhibition and co-stimulation, showed a higher score in cluster C2, whereas Type I/II IFN Response were mainly enriched in cluster C1 (Fig. 4H). As well known, the tumor immune microenvironment is tightly associated with tumor development and immunotherapeutic response, therefore, the differences in infiltrating immunocytes and immunological functions between two distinct HCC subtypes might be a key element determining the effectiveness of immunotherapy and prognosis of patients with HCC.

### Development and Corroboration of Platelet-Associated Risk Signature

**Fig. 5:**
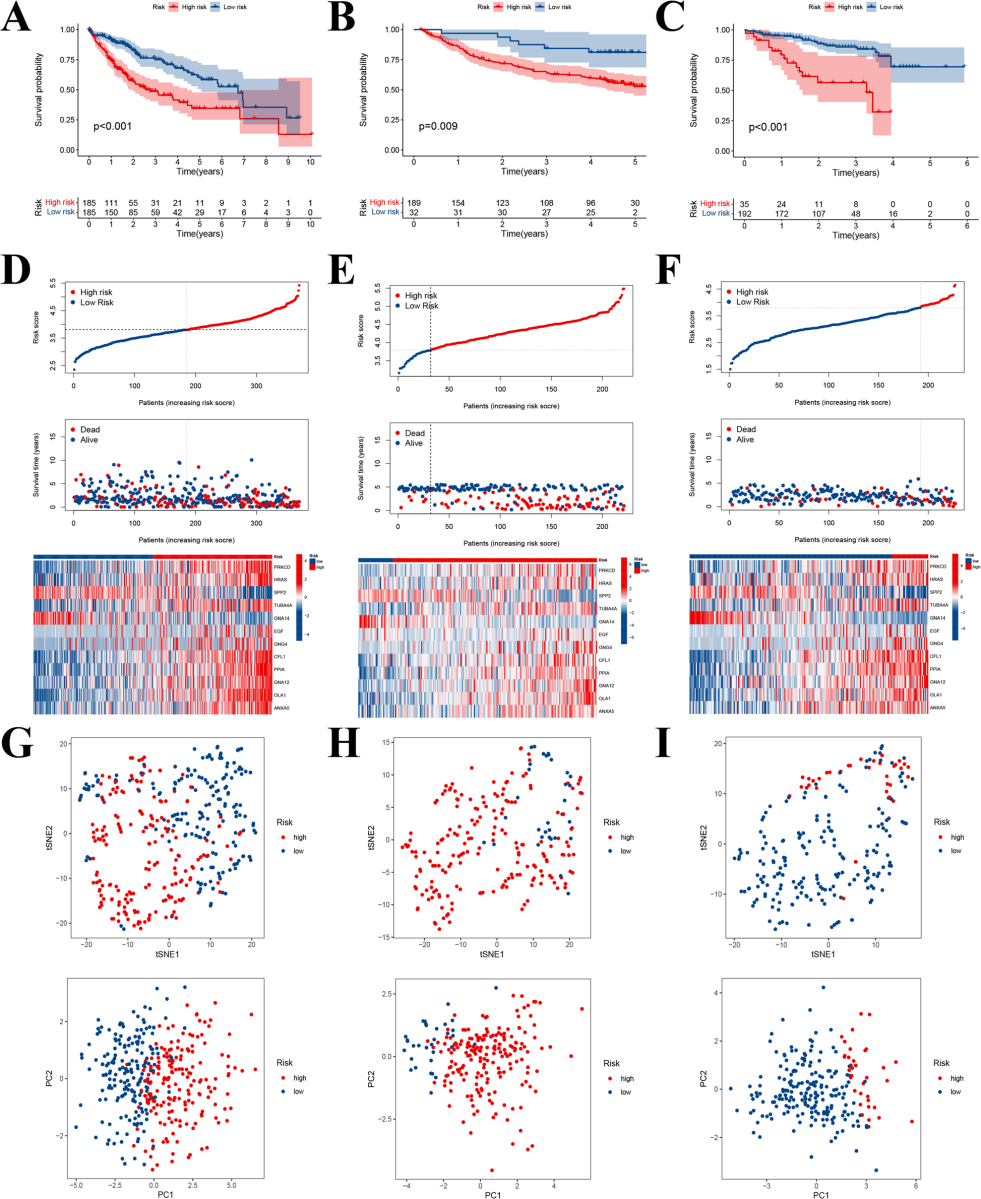
Establishment and verification of platelet-related risk model. **A** Twelve-gene included prognostic model developed via the lasso cox analysis. **B-D** The OS curves of patients in distinct risk groups that divided according to the model in the TCGA-LIHC (left), GSE14520 (middle), and ICGC-LIRI cohorts (right). **E** The risk score delamination, survival state, as well as the expression pattern of model genes in the TCGA-LIHC cohort. **F** The risk score delamination, survival state, as well as the expression pattern of model genes in the GSE14520 cohort. **G** The risk score delamination, survival state, as well as the expression pattern of model genes in the ICGC cohort. **H-J** PCA and t-SNE analyses between different risk groups in the TCGA-LIHC, GSE14520, and ICGC-LIRI cohorts

**Fig. 6:**
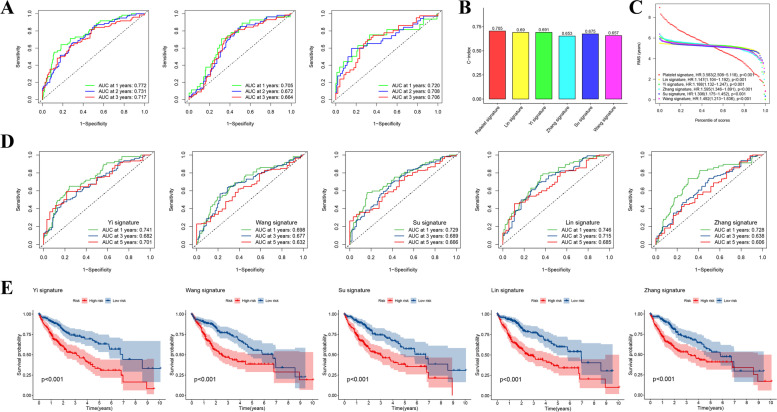
Cross-validation of predictive efficiency of the prognostic signature. **A** ROC curves of HCC patients at 1, 2, and 3 years in the TCGA-LIHC (left), GSE14520 (middle), and ICGC-LIRI (right) cohorts. **B** C-index of each risk signature: 0.705 for our platelet signature, 0.69 for Lin signature, 0.691 for Yi signature, 0.653 for Zhang signature, 0.675 for Su signature, and 0.657 for Wang signature. **C** Crosswise comparison of survival differences among distinct risk signatures based on the restricted mean survival (RMS) time: Platelet signature (HR: 3.583, 95%CI: 2.508–5.118, *p* < 0.001), Lin’s signature (HR: 1.147, 95%CI: 1.014–1.192, p < 0.001), Yi’s signature (HR: 1.188, 95%CI: 1.132–1.247, *p* < 0.001), Zhang’s signature (HR: 1.595, 95%CI: 1.346–1.891, *p* < 0.001), Su’s signature (HR: 1.306, 95%CI: 1.175–1.452, *p* < 0.001), and Wang’s signature (HR: 1.492, 95%CI: 1.213–1.836, *p* < 0.001). **D** ROC curves of HCC patients at 1, 3, and 5 years in several other published prediction models. **E** OS curves of HCC patients stratified by other published risk signatures

In this section, 29 candidate prognosis-related PRGs obtained previously were first applied to establish a risk model via the lasso cox regression analysis (Fig. S1). Then a 12 PRGs-contained risk signature was constructed according to their respective gene expression and regression coefficients as the formula indicated below: Risk Score = (0.029 * PRKCD) + (0.066 * HRAS) + (− 0.021 * SPP2) + (0.050 * TUBA4A) + (− 0.370 * GNA14) + (0.035 * EGF) + (0.066 * GNG4) + (0.109 * CFL1) + (0.028 * PPIA) + (0.231 * GNA12) + (0.150 * OLA1) + (0.080 * ANXA5) (Supplementary table: Table S3). HCC patients in the training set were subsequently classified into two distinct risk groups hinging on the median risk score (185 cases at high risk and 185 cases at low risk), KM survival curve illustrated that high-risk patients suffered a worse OS compared with low-risk ones (Fig. 5A). Moreover, similar results were further corroborated in the external datasets that the OS of high-risk patients both in the GSE14520 and ICGC-LIRI cohorts was significantly inferior to those at low risk (Fig. 5B-C). And the differences in risk scoring distribution, survival status, and risk gene expression schema between distinct risk groups within the respective cohorts were also described in Fig. 5D-F. By the way, the representative IHC staining results of these risk genes both in the normal and HCC tissues obtained from the online HPA database also proved their expression patterns from the protein level (Fig. S2). Additionally, t-SNE as well as PCA analyses also indicated that our prognostic model could effectively distinguish patients from distinct risk groups both in the training and external validation cohorts (Fig. 5G-I). To estimate the efficiency of the risk signature in prognosis prediction, we counted the AUCs in the 1-, 2-, and 3-year ROC curves respectively. The results showed that the year-standardized AUCs in the TCGA-LIHC cohort were 0.772, 0.731, and 0.717, which were 0.705, 0.672, and 0.664 in the GSE14520 cohort, as well as 0.720, 0.708, and 0.706 in the ICGC-LIRI cohort, respectively (Fig. 6A), implying that our risk signature had good accuracy and stability in prognosis assessment. Meanwhile, we also made a comparison between our risk signature with other published prediction models, including Yi’s signature [12], Wang’s signature [13], Su’s signature [14], Lin’s signature [15], and Zhang’s signature [16]. The concordance index (C-index) was used to appraise the predictive capability of distinct risk signatures, and the restricted mean survival (RMS) time was applied to compare survival differences among groups distinguished by different risk models. It was observed that our risk signature had a higher C-index and a more noteworthy survival difference compared to other signatures, indicating that our prediction model also had some advantages in horizontal comparison (Fig. 6B-C). Besides, both the 1-, 3-, and 5-year ROC curves as well as survival curves of these contrastive risk signatures were also displayed respectively (Fig. 6D-E).

### Clinicopathological Parameters Correlation Analysis

**Fig. 7:**
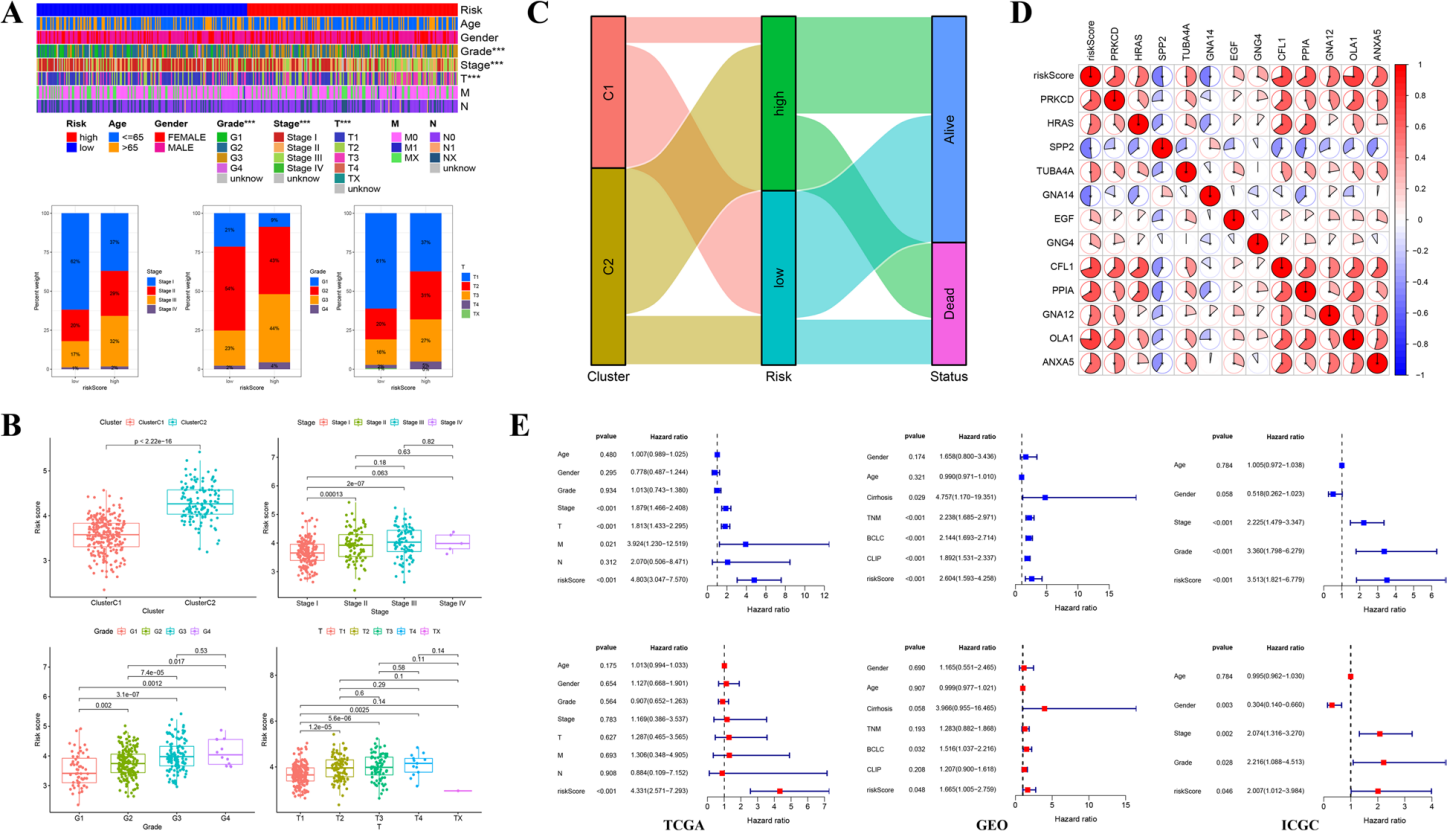
Clinical significance of the risk signature. **A** Association between clinicopathological parameters and risk scores in the TCGA-LIHC cohort (upper), percentage of tumor stage, pathological grade, and T stage between different risk groups (below). **B** Boxplots of distribution of risk scores in patients with distinct clusters, tumor stage, pathological grade, as well as T stage in the TCGA-LIHC cohort. **C** Sankey diagram displayed the potential liner link among the cluster, risk status, and patients’ survival state. **D** Correlation matrix among risk genes in the prognostic model. **E** The risk score calculated by the regression coefficient of each model gene was demonstrated to be an independent hazard factor for patients’ prognosis both in the TCGA-LIHC, GSE14520, and ICGC-LIRI cohorts. (*p < 0.05; ***p < 0.001)

Subsequently, to explore the prognostic value of our signature in patients with different clinical characteristics, we further analyzed whether there were significant connections between risk groups and clinical features. The results illustrated that the high-risk score was closely correlated with worse tumor stage (Stage II-III), T stage (T2–3), and pathological grade (G3) (Fig. 7A). The levels of pathological grade and stage increased with the elevation of risk scores, meanwhile, a conspicuous connection was also observed between risk scores and HCC clusters that cluster C2 with poor clinical outcome was markedly associated with the high-risk score (Fig. 7B), and the linear relationship among clusters, risk groups, and patients’ statuses was described with Sankey plot in Fig. 7C. In addition, the survival curves of patients with different clinical features in both risk groups were shown in Fig. S3. And the association between risk scores and clinicopathological parameters in the GSE14520 and ICGC-LIRI cohorts was also analyzed and displayed in Fig. S4. Additionally, the correlation among these risk genes both in the training and the other two external cohorts was displayed in Fig. 7D and Fig. S5. Subsequently, both uni- and multi-cox analyses were performed to assess the independent prognostic efficacy of risk scores, uni-cox analysis in the training set disclosed that both risk score and several other variables (tumor stage, T /M stage) were hazard factors for the poor prognosis, and multi-cox analysis further proved the ability of the risk score as an independent hazard factor (*p* < 0.001, HR = 4.331, 95% CI: 2.571–7.293). Meanwhile, in the GSE14520 cohort, both BCLC stage (*p* = 0.032, HR = 1.516, 95% CI: 1.037–2.216), as well as the risk score (*p* = 0.048, HR = 1.665, 95% CI: 1.005–2.759) were demonstrated to be independent hazard factors via the multi-cox analysis, the analogous results were also discovered in the ICGC-LIRI cohort that the risk score (*p* = 0.046, HR = 2.007, 95% CI: 1.012–3.984), tumor stage (*p* = 0.002, HR = 2.074, 95% CI: 1.316–3.270), and pathological grade (*p* = 0.028, HR = 2.216, 95% CI: 1.088–4.513) were verified to be independent hazard factors, while gender (*p* = 0.003, HR = 0.304, 95% CI: 0.140–0.660) was thought as a protective factor for patients’ OS (Fig. 7E). In a word, these findings indicated that our 12-gene-included risk signature was closely associated with clinical characteristics, which also had a fine predictive capacity and was expected to act as a potential prognostic indicator for HCC patients.

### Functional Analysis Based on Platelet-Related Risk Signature

**Fig. 8:**
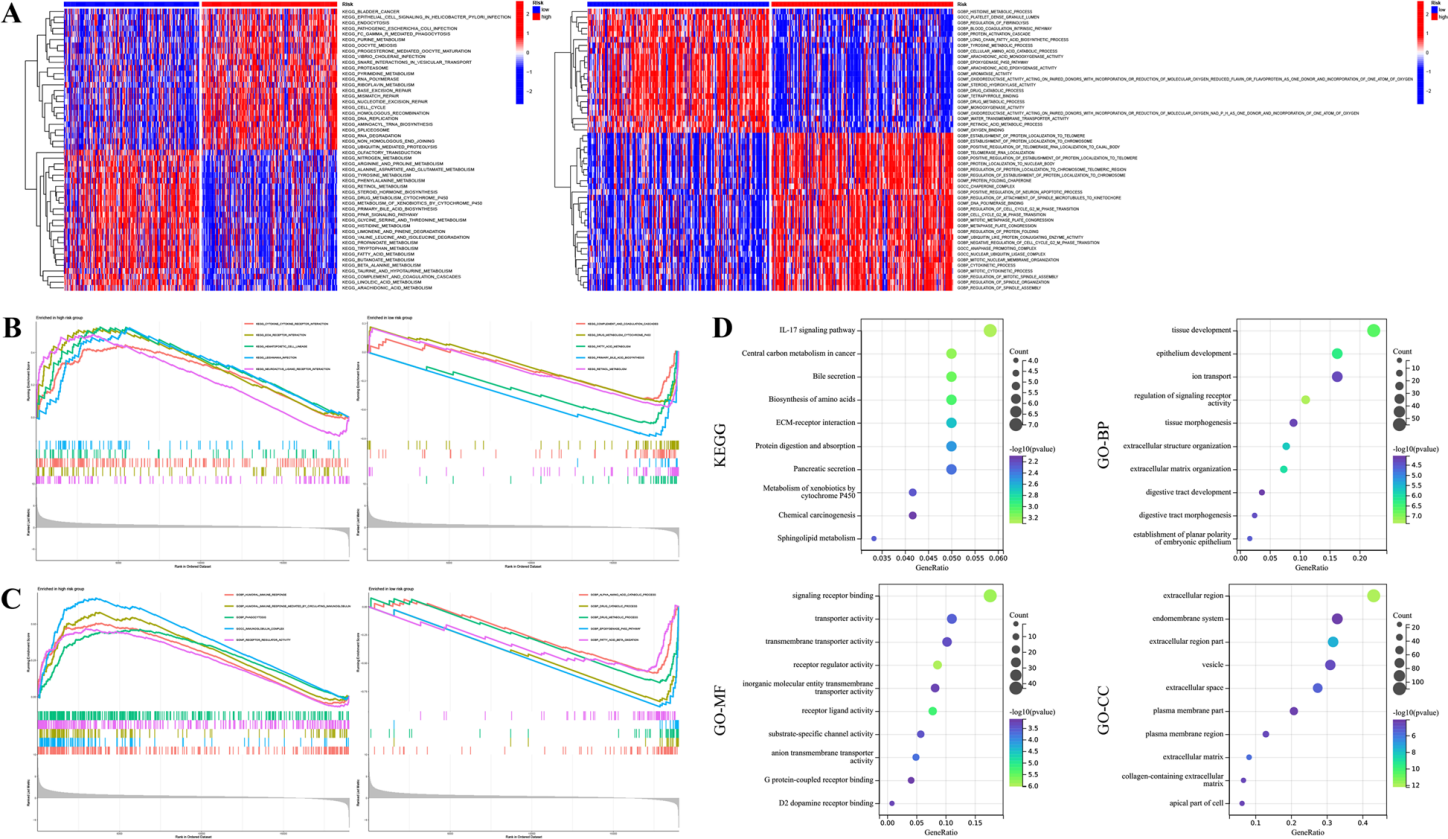
Functional enrichment analysis between distinct risk groups in the TCGA-LIHC cohort. **A** GSVA revealed the discrepancies in GO terms and KEGG pathways between different risk groups based on the platelet-related risk signature. **B** The respective enriched KEGG pathways in both risk groups according to the GSEA method. **C** The respective enriched GO terms in both risk groups according to the GSEA method. **D** Bubble plots of KEGG pathways as well as GO terms enriched by differentially expressed genes (|log_2_FC| > 2 and *p* < 0.01) between two distinct risk groups based on the platelet-related risk signature

Considering the potential discrepancies in biological processes and functional pathways between distinct risk groups in the TCGA-LIHC cohort, we exerted both GSVA and GSEA analyses to make an individualized investigation. The GSVA (GO part) results illustrated that the conspicuously enriched GO terms in patients at high risk were “regulation of protein localization and folding”, “regulation of cell cycle G2-M phase transition”, and “mitotic process” in BP, “DNA polymerase binding” and “ubiquitin-like protein conjugating enzyme activity” in MF, and “anaphase-promoting complex” in CC, implying that high-risk score was tightly connected with cell cycle and mitotic process, which might be implicated in the mediation of tumorigenesis and progression. While the primary GO terms enriched in patients at low risk included “metabolic-related process”, “coagulation and fibrinolysis process” in BP, “enzymatic activity” in MF, and “platelet dense granule lumen” in CC, indicating that low-risk score was tightly connected with platelet and metabolic related processes. Moreover, several noteworthy KEGG pathways like “cell cycle”, “DNA replication”, “RNA degradation”, as well as “ubiquitin-mediated proteolysis” were significantly concentrated in the high-risk group, by contrast, multiple metabolic-associated processes like “nitrogen metabolism”, “amino acid and lipid-related metabolism”, and “drug metabolism cytochrome P450” were primarily concentrated in the low-risk one (Fig. 8A). Subsequently, GSEA analysis was used to validate the biological annotation obtained above. It was observed that the major KEGG pathways concentrated in patients at high risk were “cytokine-cytokine receptor interaction”, “ECM receptor interaction”, and “neuroactive ligand-receptor interaction”, whereas “complement and coagulation cascades”, “drug metabolism cytochrome P450”, as well as “fatty acid metabolism” were mainly concentrated in those at low risk (Fig. 8B). GO analysis indicated that the top terms correlated with high-risk scores were “humoral immune response”, “phagocytosis”, and “immunoglobulin complex”, while “alpha amino acid catabolic process”, “drug metabolic process”, and “epoxygenase P450 pathway” were the main terms correlated with low-risk scores (Fig. 8C). Additionally, 328 DEGs were screened out between two risk groups with the threshold |log_2_FC| > 2 and *p* < 0.01, and GO and KEGG analyses were also employed according to these DEGs and the results were displayed in Fig. 8D. Similarly, the results of both GSEA and GO/KEGG analyses in the GSE14520 and ICGC-LIRI cohorts were also shown in Fig. S6.

### Investigation of Immune Characteristics

**Fig. 9:**
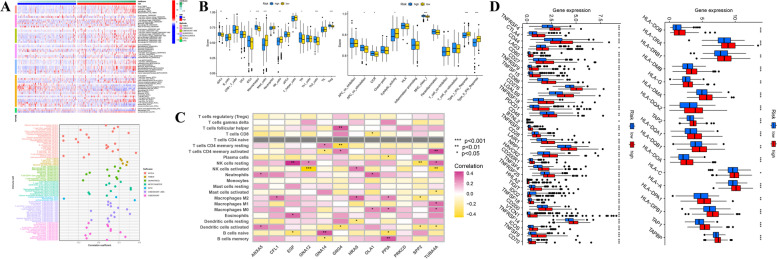
Immune landscape of patients in the TCGA-LIHC cohort classified by platelet-related prognostic signature. **A** Heatmap (left) and lollipop plot (right) of differences in infiltrating immunocytes between distinct risk groups. **B** Comparison of scores of immunocytes as well as immunological functions between two groups based on the ssGSEA algorithm. **C** Correlation matrix between model genes and infiltrating immunocytes. **D** Boxplots of the discrepancies in the expression of immune checkpoint and human leukocyte antigen (HLA) genes between distinct groups. (*p < 0.05; **p < 0.01; ***p < 0.001)

According to the immunological landscape depicted by multiple immune algorithms, an observable discrepancy in infiltrating immunocytes between distinct risk groups was discovered that the infiltrating level of immunocytes was positively associated with the risk score (Fig. 9A). And ssGSEA method-based immune analysis further confirmed that most immunocytes (including aDCs, iDCs, Macrophages, Tfh and Th2 cells, as well as Tregs) were calculated with a higher infiltrating score in patients at high risk, inversely, other immunocytes like Mast cells and NK cells displayed a higher infiltration level in those at low risk. With regard to immunological functions, such as “APC co-stimulation”, “CCR”, as well as “MHC class I” were linked to high-risk scores, while functions like “cytolytic activity”, and “Type I/II IFN Response” were linked to low-risk scores (Fig. 9B). These findings were generally consistent with the consequences obtained in the previous clustering analysis, implicating that these observations acquired in both subgroup analyses were convincing to an extent. Additionally, the correlation coefficients between risk PRGs and immunocytes were calculated respectively and described in Fig. 9C. And the discrepancies in infiltrating immunocytes or immunological functions in the other two validation cohorts were also investigated and exhibited in Fig. S7. Furthermore, the discrepancies in the expression level of immune checkpoint genes, as well as HLA genes which were connected with antigen presentation and immune response, were also displayed in Fig. 9D. Nearly all of these genes showed a higher expression level in the high-risk group, indicating that there might be a potential difference in immune status of two different groups distinguished by individual risk scores.

### Genetic Mutation and Drug-Sensitive Analysis

**Fig. 10:**
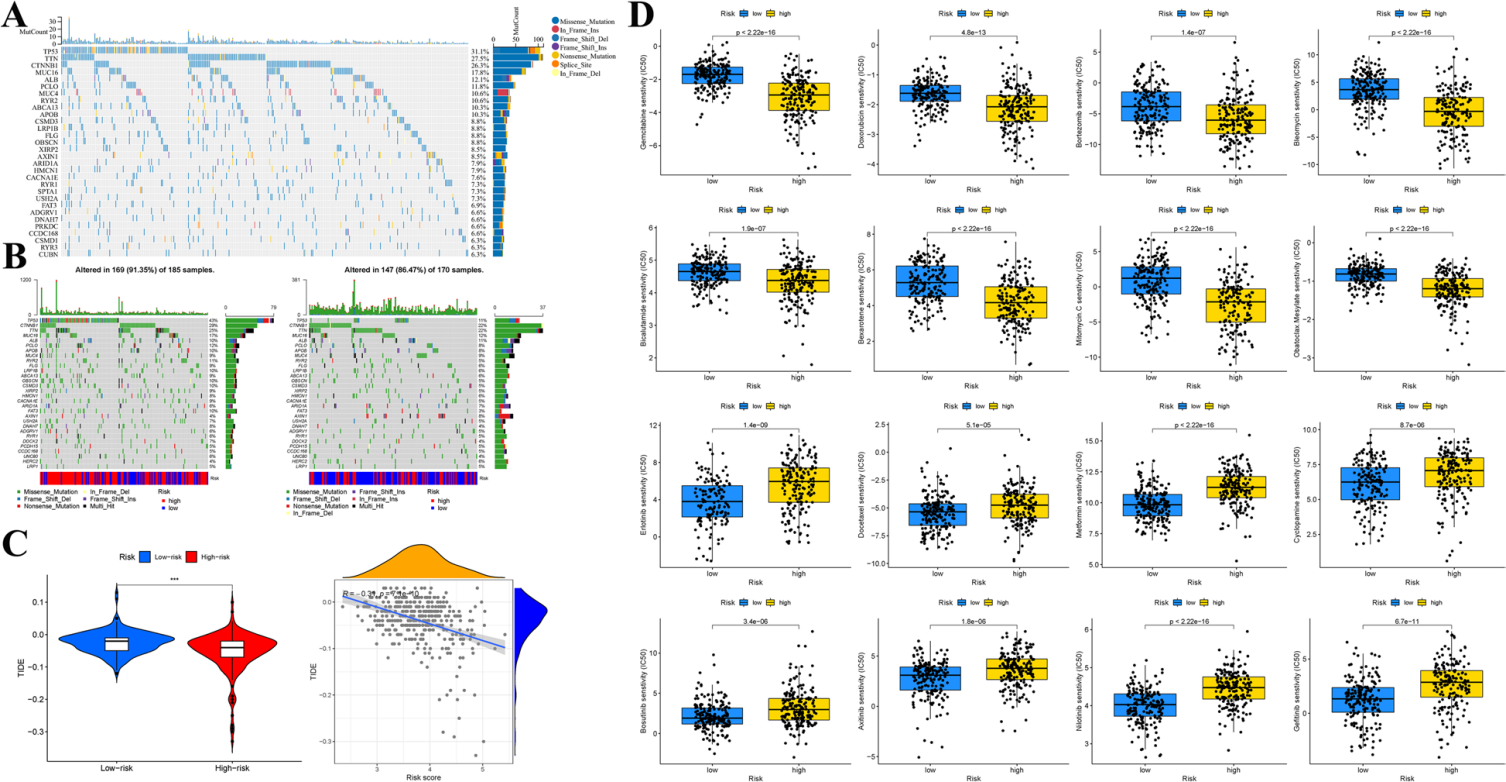
Tumor mutation and drug sensitivity analyses of patients at different risk statuses. **A** Genetic mutation landscape in the entire TCGA-LIHC cohort, with the top 30 genes displayed in the waterfall plot. **B** Waterfall plots showed the genetic mutation features both in the high- (left) and low-risk (right) groups. **C** Violin plot of the comparison of the TIDE scores between distinct risk groups (left) and their liner dependence (right). **D** Sensitivity prediction of chemotherapeutic regents in different groups. (***p < 0.001)

Considering the potential value of somatic mutations in tumor progression and individual clinical treatment, we analyzed and depicted the somatic mutation landscape of patients in the TCGA-LIHC cohort in whole and different risk groups, separately. Waterfall plots respectively displayed the somatic mutation feature of patients in the entire cohort (Fig. 10A), as well as those in two distinct risk groups (Fig. 10B). Briefly speaking, patients at high risk were demonstrated to be accompanied by a higher mutation frequency compared with low-risk ones, and missense mutation was the most frequent mutation pattern in two risk groups. Specifically, TP53 with missense mutation was markedly frequent in patients at high risk, whereas AXIN1 with frameshift insertion was relatively common in patients at low risk, respectively.

TIDE, represents tumor immune dysfunction and exclusion, is an algorithm usually employed to evaluate the possibility of immune escape and then make a prediction of immunotherapy response [17]. Here, we explored the relationship between the risk score and TIDE score to investigate the potential significance of the risk model in immunotherapy prediction. The results indicated that there was an obvious negative association between two variables (R = − 0.31, *p* = 7.1e^− 10^), patients at low risk had a higher TIDE score, implying that our risk signature possessed the capacity of immunotherapy prediction and patients at low risk might be more susceptive to immunotherapeutic compared with high-risk patients (Fig. 10C). Subsequently, the correlation between risk scores and IC_50_ values of frequently used chemotherapeutic medicaments was analyzed to explore the feasible sensitive agents. As Fig. 10D showed, a lower IC_50_ value of gemcitabine, doxorubicin, bortezomib, bleomycin, bicalutamide, bexarotene, mitomycin C, and obatoclax mesylate was observed in the high-risk group, implicating that patients at high risk might benefit from these chemotherapeutic agents, while patients at low risk might profit from the following drugs, including erlotinib, docetaxel, metformin, cyclopamine, bosutinib, axitinib, nilotinib, and gefitinib.

### In Vitro Functional Verification

**Fig. 11:**
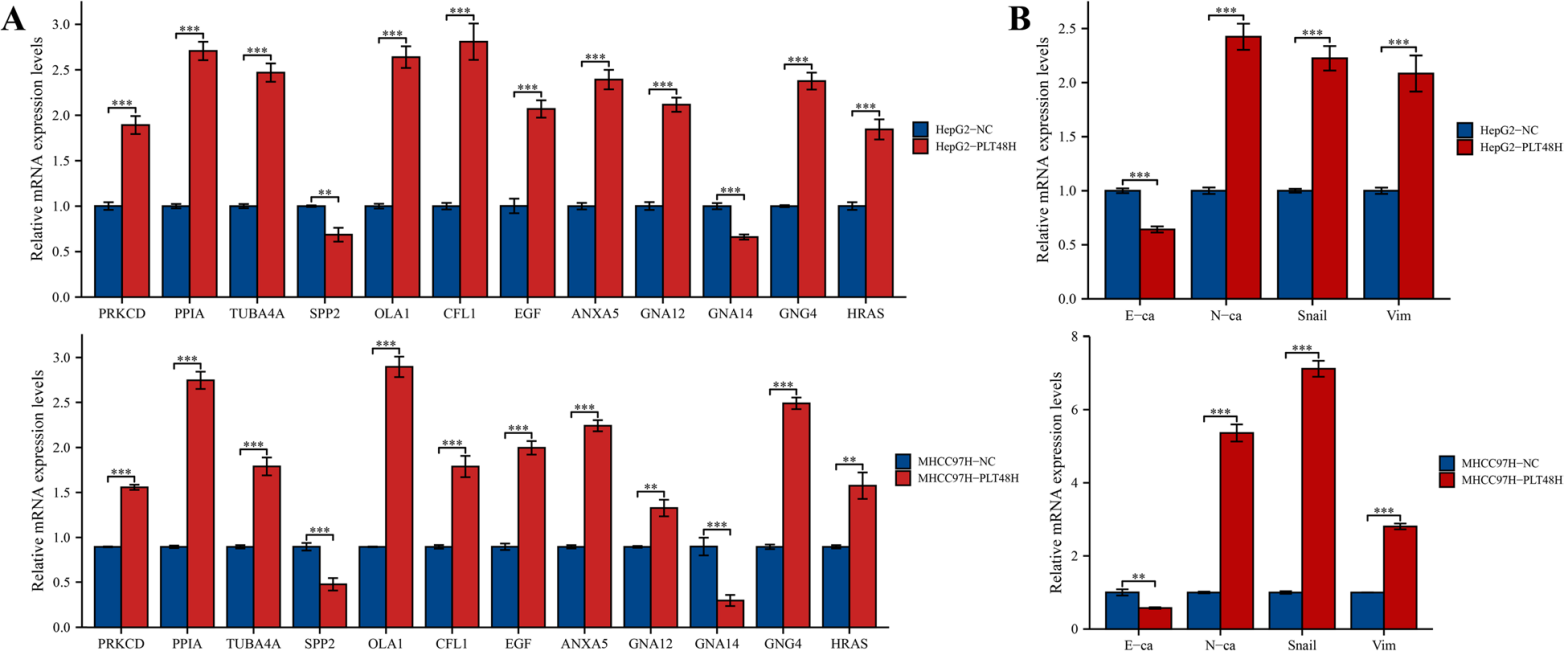
**A** Effects of in vitro platelet stimulation on the mRNA expression levels of risk genes in HepG2 and MHCC97H cells. **B** Effects of in vitro platelet stimulation on the mRNA expression levels of EMT-related biomarkers in HepG2 and MHCC97H cells. (**p < 0.01; ***p < 0.001)

**Fig. 12:**
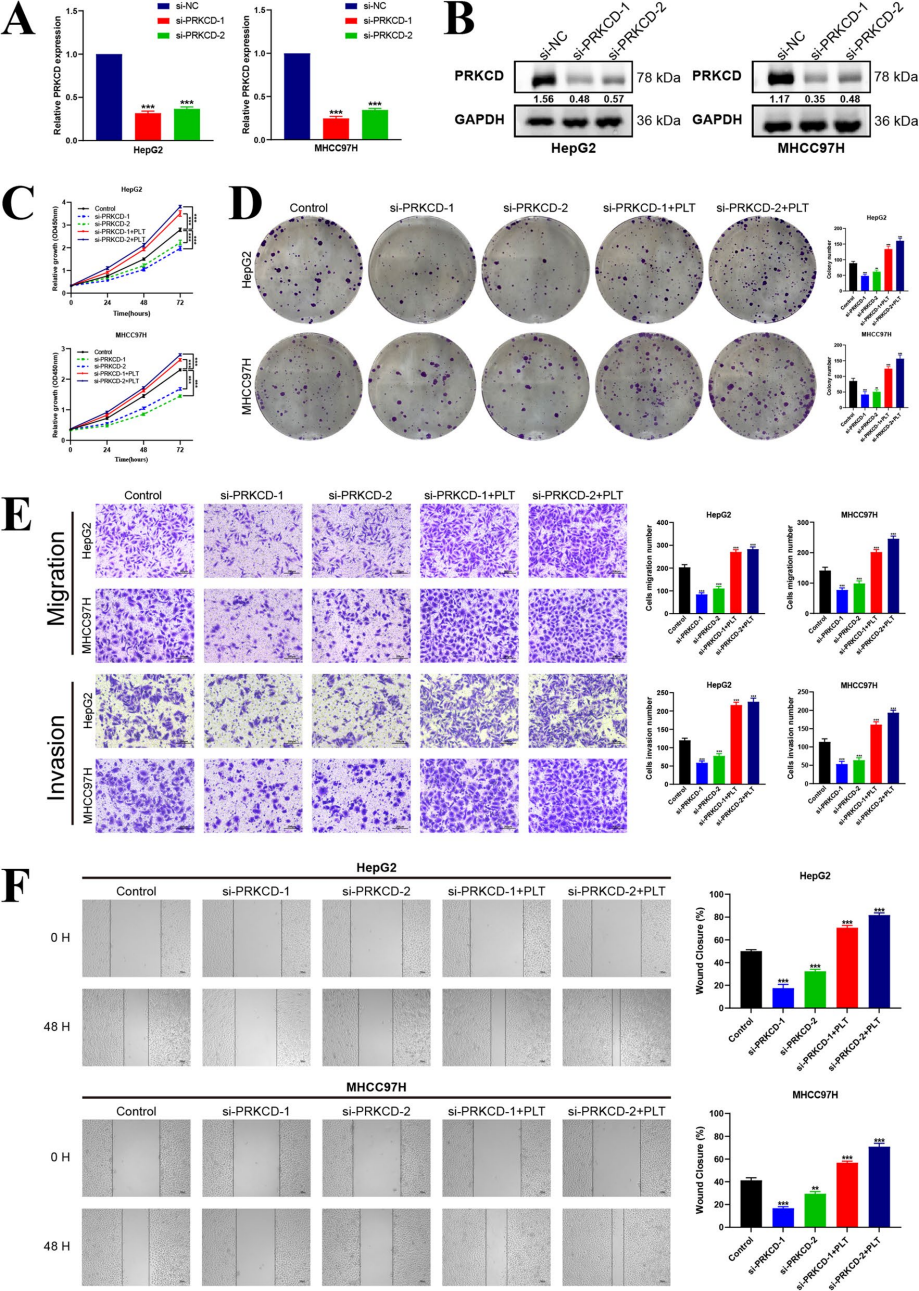
In vitro validation of PRKCD on HCC cells proliferation, invasion and migration. **A-B** Measurement of both mRNA and protein expression levels of PRKCD in HepG2 and MHCC97H cells transfected with two PRKCD siRNA sequences, si-NC was utilized as the negative control. **C** Effects of si-PRKCD and platelet stimulation on proliferation abilities of HepG2 and MHCC97H cells measured by the CCK-8 assay. **D** Effects of si-PRKCD and platelet stimulation on proliferation abilities of HepG2 and MHCC97H cells measured by the colony formation test. **E** The transwell assay was performed to assess the impacts of si-PRKCD and platelet stimulation on HCC cells migration (upper) and invasion (below) capacities. Scale bar: 200 μm (200 ×). **F** The wound healing test displayed the migration ability of HCC cells undergone different treatments. Scale bar: 100 μm (40 ×). The results were presented with representative images from three times independent replicate experiments, and all data were shown as Means ± SD. (**p < 0.01; ***p < 0.001)

**Fig. 13:**
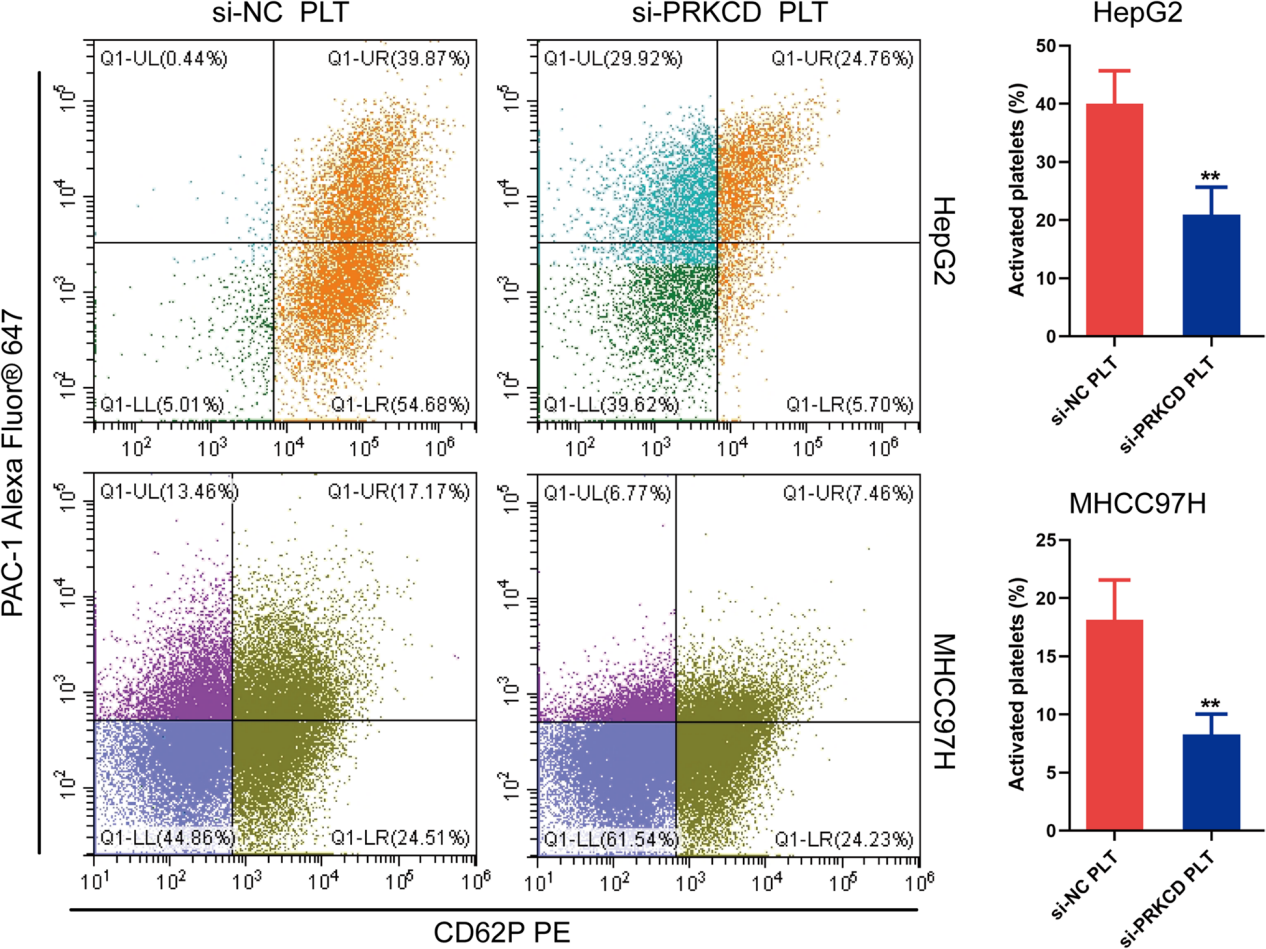
Flow cytometry detection of platelets after co-cultured with cancer cells. HepG2 and MHCC97H cells were transfected with si-NC and si-PRKCD respectively, platelets were added and co-incubated with them, and the collected platelets were stained using CD62P and PAC-1. (**p < 0.01)

To further investigate the interaction between tumor cells and platelets in vitro, the platelets from human peripheral blood were first extracted and isolated and then co-cultured with HCC cells (HepG2 and MHCC97H) for 48 h. As Fig. 11A exhibited**,** the consequences of RT-qPCR demonstrated that the mRNA expression levels of PRKCD, HRAS, TUBA4A, EGF, GNG4, CFL1, PPIA, GNA12, OLA1, and ANXA5 were distinctly elevated after platelet stimulation, whereas the expression of SPP2 as well as GNA14 displayed a significant decreasing trend, which was precisely consistent with the expression patterns of these risk genes in our prognostic signature. Meanwhile,

the expression of N-cadherin (N-ca), Vimentin (Vim), and Snail was also increased obviously, whereas the expression of E-cadherin (E-ca) displayed an opposite trend both in HepG2 and MHCC97H cells, implying that platelet stimulation could induce epithelial-mesenchymal transition (EMT) in HCC cells (Fig. 11B). Subsequently, considering the research-supported close relationship between PRKCD and platelets, we chose PRKCD as the target molecule for further exploration [18–21]. As Fig. 12A-B displayed, both the mRNA and protein expression levels of PRKCD were first knocked down by si-PRKCD transfection, and after 24 h transfection, platelets were added and co-cultured with tumor cells for another 48 h. Subsequently, we investigated the impacts of distinct transfection treatments (including si-NC (Control), si-PRKCD-1, si-PRKCD-2, si-PRKCD-1 + PLT, si-PRKCD-2 + PLT) on HCC cells’ vitality and motility. Both the CCK-8 assay and colony formation test illustrated that the proliferative vitality of two HCC cells was significantly restrained by PRKCD silencing, and analogously, this inhibitory influence could be corrected by platelet stimulation (Fig. 12C-D). Moreover, the transwell assay illustrated that the invasion and migration capabilities of HepG2 as well as MHCC97H cells were markedly suppressed by PRKCD silencing, which could be reversed by direct co-culture with platelets (Fig. 12E). And the wound healing test further demonstrated that the reduction of PRKCD could obviously attenuate the migration capacity of HepG2 and MHCC97H cells and this inhibitory influence was apparently reversed by the supplement of platelets (Fig. 12F). These findings indicated that platelet stimulation could up-regulate the expression of PRKCD, which was further confirmed to be involved in mediating tumor malignant phenotypes through in vitro experiments, implicating that PRKCD was involved in platelet-induced HCC progression. While as a positive feedback loop, whether PRKCD could affect platelet activation remained unclear. Here, we hypothesized that the expression level of PRKCD in HCC cells could affect the activation of platelets in vitro. To confirm our assumption, the expression level of cellular PRKCD was first suppressed by si-PRKCD transfection (si-PRKCD-1 with better inhibition performance was selected in this section), and the PRKCD-silencing HCC cells were then utilized to stimulate the isolated platelets by direct contact, eventually, the activation level of platelets was analyzed by flow cytometric assay. The results demonstrated that PRKCD-silencing HCC cells could effectively inhibit the activation level of platelets compared to the negative control (Fig. 13), which was a confirmation of our conjecture. In summary, these results preliminarily proved that our risk gene PRKCD could either participate in the modulation of malignant biological behaviors of cancer cells as well as regulate the activation of platelets in vitro, implicating that PRKCD might act as a key molecular bridge in the cross-talk between HCC cells and platelets and also take an essential part in the platelet-induced HCC progression. Meanwhile, to explore whether platelet stimulation can mediate HCC progression by regulating the expression levels of other risk genes, based on the previous literature reports [22, 23], we selected another two risk genes (ANXA5 and CFL1) that might be related to platelets for subsequent in vitro functional verification. And based on previous studies of these two genes in HCC [24, 25], we synthesized two siRNA sequences respectively to knockdown the expression level of ANXA5 and CFL1 to investigate their potential function. In vitro functional experiments indicated that ANXA5 was involved in the regulation of HCC cells proliferation, migration, and invasion, and knockdown of its expression could inhibit the malignant phenotype of HCC (Fig. S8), and similar results were also observed after CFL1 knockdown treatment (Fig. S9). These findings are consistent with the hypothesis that platelets may mediate the malignant progression of HCC by regulating the expression of the above risk genes. However, the results of flow cytometry showed that intervention with ANXA5 or CFL1 had no significant effect on platelet activation, implying that these two genes may not be involved in the regulation of HCC-mediated platelet activation.

## Discussion

Liver cancer refers to a type of fatal digestive system malignancy that seriously threatens public health around the world. Based on the recent global cancer statistics, the number of new incidents (905,677, 4.7%) and deaths (830,180, 8.3%) of patients with liver malignancy occupied sixth place in terms of cancer-related incidence and third place in mortality around the world, respectively [1]. As for the tumor burden in China, recent statistics showed that the new liver cancer cases diagnosed in 2016 were 388,800, the male was 288,800 persons and female was 100,000 persons. In terms of disease-related deaths, a total of 336,400 persons (249,600 men and 86,800 women) died of liver cancer in 2016. Generally speaking, its morbidity and mortality (28.12 and 24.33 per 100,000) ranked fourth and second place in cancer-related big data in China, respectively [2]. In the past period of time, with the in-depth understanding of the hazard factors related to hepatocarcinoma, as well as its popularization of prevention strategies and advances in comprehensive treatment measures, the overall incidence and mortality rates of hepatocarcinoma in China showed a decreasing trend, yet these rates displayed a sustaining rising trend in the United States and several European regions over the past few decades [2, 26]. Therefore, liver cancer remains an important public health problem that endangers the national economy and life health, which needs people’s constant attention and exploration of its epidemiology and internal pathological mechanisms.

A holistic recognition of epidemiological risk factors (represented by chronic viral hepatitis and liver cirrhosis) and internal molecular mechanisms of tumor occurrence and development is of pivotal significance for the prevention and management of HCC. Generally, the ultrasound examination and serum alpha-fetoprotein (AFP) levels are widely utilized for the early screening of high-risk populations, and the suspected patients subsequently obtain a clinical or definitive diagnosis via the CT/MRI enhanced scanning or liver biopsy. In recent years, several commonly used blood routine and biochemistry indexes have also attracted much attention from researchers in addition to the classic serum oncology biomarkers, which have the potential to serve as prospective indicators for prognosis assessment and risk stratification of patients with HCC independently or synergistically [27–29], and platelet is one of the representatives [11, 30–32]. Studies have demonstrated that abnormal platelet counts (including thrombocytopenia and thrombocytosis) are closely related to the poor OS of patients with HCC [30, 33]. Additionally, aberrant changes in morphology or functional status of platelet have also been discovered to be connected with tumor metastasis and patients’ adverse outcomes [11, 31]. Actually, the intimate connection between platelets and tumor progression has been widely recognized, numerous fundamental research has proved that platelets take an essential part in mediating cancer proliferation, angiogenesis, invasion and metastasis, as well as immune escape [4]. Concerning HCC, it has been confirmed that the platelet-derived growth factors (PDGF, including PDGFA, PDGFB, PDGFC, and PDGFD) participate in modulating the tumor development and chemoresistance by interacting with its receptor PDGFR and activating the downstream signaling pathway [34–36]. Besides the PDGF family, other cytokines secreted by platelets have also been proved to be associated with HCC. For example, serum vascular endothelial growth factor (VEGF, primarily from platelets) was shown to be directly connected with the OS of HCC patients, including both primary and treated cancer patients [37, 38]. Mechanistically, the VEGF signaling pathway has been widely reported to be connected with angiogenesis in HCC and is involved in the regulation of tumor progression [39]. Besides, transforming growth factor beta 1 (TGF-β1) can also be discharged from platelets, which represents the major source of peripheral circulating TGF-β1 that plays a multifaceted role in mediating tumor development [8]. Considering the important role of the direct or indirect interaction between platelets and tumor cells in the regulation of tumor progression, anti-platelet therapeutics that target platelet receptors or inhibit the activity of related enzymes (like aspirin and clopidogrel) also occupy an essential part in cancer treatment [4]. To sum up, platelet refers to the largest and most widespread circulatory repository of tumor-promoting factors (such as PDGF, VEGF, and TGF-β1) in the human body, the research on it is of great clinical significance, especially in cancers with abundant blood distribution, like HCC.

In our study, we collected a total of 300 PRGs and performed clustering analysis on HCC patients based on the PRGs associated with prognosis. And on this basis, a 12-gene prognostic model was further developed through the lasso cox analysis, comprising PRKCD, HRAS, SPP2, TUBA4A, GNA14, EGF, GNG4, CFL1, PPIA, GNA12, OLA1, and ANXA5. As for the potential connections between these genes and HCC, briefly speaking, the PRKCD signaling axis is tightly associated with the malignant phenotype of HCC, which can be attributed to its phosphorylation kinase activity and the activation of downstream oncogenic signaling pathways (like MAPK signaling pathway) [40]. Oppositely, several studies have also reported its suppressive functions in HCC, which hinges on the intracellular p53 status [41, 42]. HRAS, a member of the RAS gene family that is closely related to multiple human cancers, and studies have shown that it engages in mediating EMT of HCC cells through the TGF-β1/HRAS axis, thereby promoting tumor invasion and metastasis [43]. And the expression of GNA14 is decreased in tumor tissues and is related to the poor OS of patients with HCC, indicating that it can be a tumor suppressor gene. Recent research has demonstrated this view that GNA14 inhibits HCC progression by interacting with RACK1 and attenuating MAPK/JNK and PI3K/AKT axis [44]. About EGF, numerous studies have demonstrated its capacity of mediating the malignant biological behavior of HCC is reached through interacting with EGF receptor (EGFR) and activating specific oncogenic signaling pathways, like ERK and PI3K axis [45, 46]. And in the hypoxic tumor microenvironment of HCC, CFL1 can be transcriptionally activated by hypoxia-inducible factor-1α (HIF-1α), and subsequently interacts with PLD1 to inhibit PLD1 ubiquitination degradation and stabilize its expression, thereby activating the AKT pathway [24]. The expression levels of PPIA are often up-regulated in tumor specimens and markedly associated with tumor size, which may take a crucial part in modulating the proliferative ability of HCC cells [47]. And GNA12 (namely Gα12) induces zinc-finger E-box-binding homeobox (ZEB1) generation by decreasing p53-responsive miRNAs (like miR-200a/b, miR-192, and miR-215), thus facilitating EMT of HCC [48]. OLA1 has been reported to be connected with cell cycle as well as apoptosis in HCC, which promotes tumor development by binding with p21 and stimulating the expression of CDK2, knocking down its expression contributes to G0/G1 phase arrest and enhancing apoptosis of cancer cells [49]. Finally, ANXA5 refers to a calcium-dependent phospholipid-binding protein that acts as an oncogene in HCC, which enhances the invasive growth of HCC via the integrin and MEK/ERK approaches [25]. However, relevant studies about the role of the residual risk genes (including SPP2, TUBA4A, and GNG4) remain insufficient in HCC, and further investigation is requisite to explore their potential functions.

After constructing the platelet-related risk signature, we subsequently performed multiple biological information analyses to investigate its significance in HCC. Separately, HCC patients were classified into different risk groups based on the median risk score calculated by the corresponding algorithm, and patients at high risk suffered a poorer prognosis than those at low risk, and the ROC curves also proved the accuracy of the risk signature in prognosis prediction, these findings were further verified in two independent HCC cohorts, implicating that our risk signature was stable and reliable. Meanwhile, we also investigated the initial connection between risk scores and clinical features, and it was observed that the high-risk score was usually accompanied by worse clinical characteristics. Moreover, the results of enrichment analyses (including GSEA, GSVA, and ssGSEA) also revealed the differences in biological function and immune microenvironment composition among patients in different risk groups, which might contribute to further exploration of intervention targets and guiding individual treatment strategies.

Considering the risk signature was correlated with PRGs, we further validated our model by co-culturing tumor cells and platelets in vitro. The results showed that platelets stimulation could significantly enhance the invasion and migration ability of cancer cells, and what was exciting was that the transcription levels of risk genes also displayed corresponding changes after co-culture, indicating that platelets might modulate the expression of these genes by direct contact or secretion of specific cytokines, thus promoting HCC progression. Consequently, we then investigated whether there were direct causal links between these genes and platelets, and it was interesting that we found a close relationship between PRKCD and platelets. The research demonstrated that PRKCD participated in regulating megakaryocyte maturation and platelets generation [50], and the phosphorylation of PRKCD and the activation of downstream signaling pathways it triggered were also crucial for platelet activation and aggregation [51], implying that PRKCD acted as an important mediator in platelet signaling and function modulation. And at the same time, in breast cancer, PRKCD could significantly enhance the invasiveness of tumor cells in vitro, which was tightly related to the activation of PRKCD after platelets stimulation and the up-regulation of MMP-9 induced by it, indicating that PRKCD played a pivotal role in mediating the tumor promotion after incubation with platelets, and targeting PRKCD to cut off the interaction between platelets and tumor cells was expected to be a potential therapeutic strategy for breast cancer [52]. Combined with the above research findings, we hypothesized whether PRKCD also served as an essential transductor in mediating platelet-induced HCC invasion and metastasis. To confirm this hypothesis, we first demonstrated that co-culture with platelets could activate the transcriptional level of PRKCD in HCC cells via RT-qPCR assay. Then we knocked down the expression of intracellular PRKCD and found that PRKCD silencing could significantly suppress the proliferation, invasion, and migration abilities of cancer cells. Subsequently, PRKCD knock-down HCC cells were further incubated with platelets and the results showed that the attenuation of proliferation or invasion abilities caused by PRKCD inhibition could be markedly reversed by co-culture with platelets. These findings provided cogent experimental evidence for our conjecture that PRKCD was indeed involved in mediating the platelet-induced malignant phenotype of HCC. Next, we collected HCC cells under different treatment conditions and separately stimulated platelets by direct contact to investigate whether PRKCD-silencing HCC cells could affect platelet activation. The consequence of flow cytometric analysis demonstrated that the activation level of platelets was significantly reduced after stimulation by PRKCD-silencing HCC cells compared with the negative control (HCC cells transfected with si-NC), implying that PRKCD in tumor cells may influence platelet activation, while the specific molecular mechanism still needs to be further investigated.

In summary, this work is the first to develop and verify a novel prognostic risk model for HCC patients according to the PRGs. Meanwhile, we conducted functional verification on PRKCD, which was most closely correlated with platelets in the risk model. The results showed that PRKCD not only modulated the malignant behaviors of tumor cells but also participated in the regulation of platelet activation, indicating that PRKCD acted as a key mediator in the cross-talk between HCC cells and platelets, and targeting PRKCD might be a hopeful intervention mean for the treatment of HCC. Nevertheless, there are still many deficiencies and limitations in our research. First, our study established a platelet-related risk signature for the prognosis evaluation of HCC patients, despite we validated our prognostic model both in the GSE14520 as well as ICGC-LIRI cohorts, massive integral HCC patient data remain needed to further confirm its applicability in clinical practice. Second, in our in vitro validation section, we only knocked down the expression of PRKCD in HCC cells to verify its biological functions but did not verify its overexpression. This was because we observed that platelet stimulation could raise the expression of PRKCD, thus we chose to down-regulate its expression level and performed a rescue experiment by adding exogenous platelets, which was also convincing to an extent. Next, we demonstrated that PRKCD reduction could suppress the activation of platelets only through the flow cytometric analysis, while the underlying mechanism was not discussed in depth, and further research was needed to investigate this. Finally, we have verified our findings through in vitro experiments, while the corresponding in vivo study is still insufficient, which is the drawback of this research and is also what we need to improve in our future work.

## Conclusion

In brief, this work reveals a novel platelet-related risk signature for prognostic evaluation of HCC patients and confirms that PRKCD is a key messenger in the interaction between HCC cells and platelets, which plays a crucial role in mediating platelet-induced tumor progression. This thesis may strengthen our cognition of platelets in the tumor microenvironment of HCC and open up novel horizons for further exploration of valuable, potential therapeutic strategies for HCC.

## Supplementary Information

**Additional file 1: Figure S1.** Lasso cox regression analysis was conducted to establish a prognostic signature including 12 platelet-related genes (optimum λ = 12).

**Additional file 2: Figure S2.** Immunohistochemistry (IHC) staining results of protein expression verification of PRKCD, HRAS, TUBA4A, CFL1, GNA12, PPIA, OLA1, ANXA5, and GNA14 between normal and HCC tissues in the HPA database. The results displayed that except for GNA14, the protein expression levels of other risk genes were significantly increased in tumor tissues, which was consistent with consistent with their expression patterns at the mRNA levels.

**Additional file 3: Figure S3.** Kaplan-Meier survival curves of patients with different clinicopathological parameters (age, gender, pathological grade, tumor stage, and TNM stage) both in the high- and low-risk groups in the TCGA-LIHC cohort.

**Additional file 4: Figure S4.** Clinical correlation analysis of HCC patients at different risk scores in the GSE14520 and ICGC-LIRI cohorts. A significant association between patients’ age and high-risk score was observed in the GSE14520 cohort, and an obviously correlation between pathological grade (G3), tumor stage (Stage III-IV), and high-risk score in the ICGC-LIRI cohort. (*p < 0.05; ***p < 0.001).

**Additional file 5: Figure S5.** Association among risk genes in the GSE14520 and ICGC-LIRI cohorts analyzed by Spearman’s correlation test. (*p < 0.05).

**Additional file 6: Figure S6.** Enrichment analysis in the GSE14520 and ICGC-LIRI cohorts. (**A**) GSEA results with top 10 KEGG pathways (such as cell cycle and cancer-related pathways enriched in the high-risk group) in the GSE14520 cohort. (**B**) GSEA results with top 10 KEGG pathways (including cell cycle, DNA replication, ubiquitin mediated proteolysis, and wnt-signaling pathways enriched in the high-risk group) in the ICGC-LIRI cohort. (**C**) GO (upper) and KEGG (below) results based on the DEGs (|logFC| > 1 and *p* < 0.01) between different risk groups in the GSE14520 cohort. (**D**) GO (upper) and KEGG (below) results based on the DEGs (|logFC| > 1 and *p* < 0.01) between different risk groups in the ICGC-LIRI cohort.

**Additional file 7: Figure S7.** Score comparison of immune cells and functions between different risk groups in the GSE14520 (**A**) and ICGC-LIRI (**B**) cohorts. (*p < 0.05; **p < 0.01; ***p < 0.001).

**Additional file 8: Figure S8.** In vitro validation of ANXA5 on HCC cells proliferation, invasion and migration, as well as platelet activation. (**A**) Measurement of mRNA expression levels of ANXA5 in MHCC97H and HepG2 cells transfected with two ANXA5 siRNA sequences, si-NC was utilized as the negative control. (**B**) Effects of si-ANXA5 on proliferation abilities of MHCC97H and HepG2 cells measured by the CCK-8 assay. (**C**) The transwell assay was performed to assess the impacts of si-ANXA5 on HCC cells migration (upper) and invasion (below) capacities. Scale bar: 200 μm (200×). (**D**) The wound healing test displayed the migration ability of HCC cells undergone different treatments. Scale bar: 100 μm (40×). (**E**) Effects of si-ANXA5 on proliferation abilities of MHCC97H and HepG2 cells measured by the colony formation test. (**F**) Flow cytometry was performed to determine the effect of knockdown ANXA5 on platelet activation in MHCC97H cell and platelet co-culture system. The results were presented with representative images from three times independent replicate experiments, and all data were shown as Means ± SD. (**p < 0.01; ***p < 0.001).

**Additional file 9: Figure S9.** In vitro validation of CFL1 on HCC cells proliferation, invasion and migration, as well as platelet activation. (A) Measurement of mRNA expression levels of CFL1 in MHCC97H and HepG2 cells transfected with two CFL1 siRNA sequences, si-NC was utilized as the negative control. (B) Effects of si-CFL1 on proliferation abilities of MHCC97H and HepG2 cells measured by the CCK-8 assay. (C) The transwell assay was performed to assess the impacts of si-CFL1 on HCC cells migration (upper) and invasion (below) capacities. Scale bar: 200 μm (200×). (D) The wound healing test displayed the migration ability of HCC cells undergone different treatments. Scale bar: 100 μm (40×). (E) Effects of si-CFL1 on proliferation abilities of MHCC97H and HepG2 cells measured by the colony formation test. (F) Flow cytometry was performed to determine the effect of knockdown CFL1 on platelet activation in MHCC97H cell and platelet co-culture system. The results were presented with representative images from three times independent replicate experiments, and all data were shown as Means ± SD. (**p < 0.01; ***p < 0.001).

**Additional file 10: Table S1.** 300 Platelet-related genes. **Table S2.** Primer sequences of genes in the risk signature. **Table S3.** 12 PRGs extracted via Lasso regression analysis.

**Additional file 11: Supplementary file 1.** The RNA-seq information of 370 HCC patients in the TCGA-LIHC cohort.

**Additional file 12: Supplementary file 2**. The corresponding survival and pathological data in the TCGA-LIHC cohort.

**Additional file 13: Supplementary file 3.** The RNA-seq information of 221 HCC cases in the GSE14520 cohort.

**Additional file 14: Supplementary file 4.** The corresponding survival and pathological data in the GSE14520 cohort.

**Additional file 15: Supplementary file 5.** The RNA-seq information of 227 cases in the ICGC-LIRI cohort.

**Additional file 16: Supplementary file 6.** The corresponding survival and pathological data in the ICGC-LIRI cohort.

## Data Availability

All datasets and materials involved in this study are provided in the article/Additional files. And the scripts utilized during this work are available from the corresponding author on reasonable request.

## Abbreviations

HCC: Hepatocellular carcinoma
LASSO: Least absolute shrinkage and selection operator
TCGA: The Cancer Genome Atlas Dataset
GEO: Gene Expression Omnibus
ICGC: International Cancer Genome Consortium
FPKM: Fragments per kilobase of transcript per million fragments mapped data
TPM: Transcripts per kilobase million values
PRGs: Platelet-related genes
GO: Gene Ontology
KEGG: Kyoto Encyclopedia of Genes and Genomes
KM: Kaplan-Meier
OS: Overall survival
PFS: Progression free survival
GSVA: Gene set variation analysis
ssGSEA: Single sample gene set enrichment analysis
PCA: Principal component analysis
ROC: Receiver operating characteristic
AUC: Area under the curve
BP: Biological process
MF: Molecular function
CC: Cellular component
TIDE: Tumor immune dysfunction and exclusion
IC_50_: Half-maximal inhibitory concentration
PPI: Protein-protein interaction
CTCC: Center for Type Culture Collection
STR: Short tandem repeat
FBS: Fetal bovine serum
DMEM: Dulbecco’s modified Eagle medium
siRNA: Small interference RNA
IHC: Immunohistochemistry
HPA: Human Protein Atlas
RT-qPCR: Real-time quantitative PCR
SDS-PAGE: Sodium dodecyl sulfate polyacrylamide gels
PVDF: Polyvinylidene fluoride
CCK-8: Cell counting kit-8
PRP: Platelet-rich plasma
SD: Standard deviation
C-index: Concordance index
RMS: Restricted mean survival
EMT: Epithelial-mesenchymal transformation
AFP: Alpha-fetoprotein
PDGF: Platelet-derived growth factor
VEGF: Vascular endothelial growth factor
TGF-β1: Transforming growth factor beta 1
HIF-1α: Hypoxia-inducible factor-1α
