# Supplementary figures and images for: Genetic Analysis of Platelet-Related Genes in Hepatocellular Carcinoma Reveals a Novel Prognostic Signature and Determines PRKCD as the Potential Molecular Bridge
Source: Biol Proced Online. 2022 Dec 3;24:22. doi: 10.1186/s12575-022-00185-9 (PMC9719151; doi:10.1186/s12575-022-00185-9)

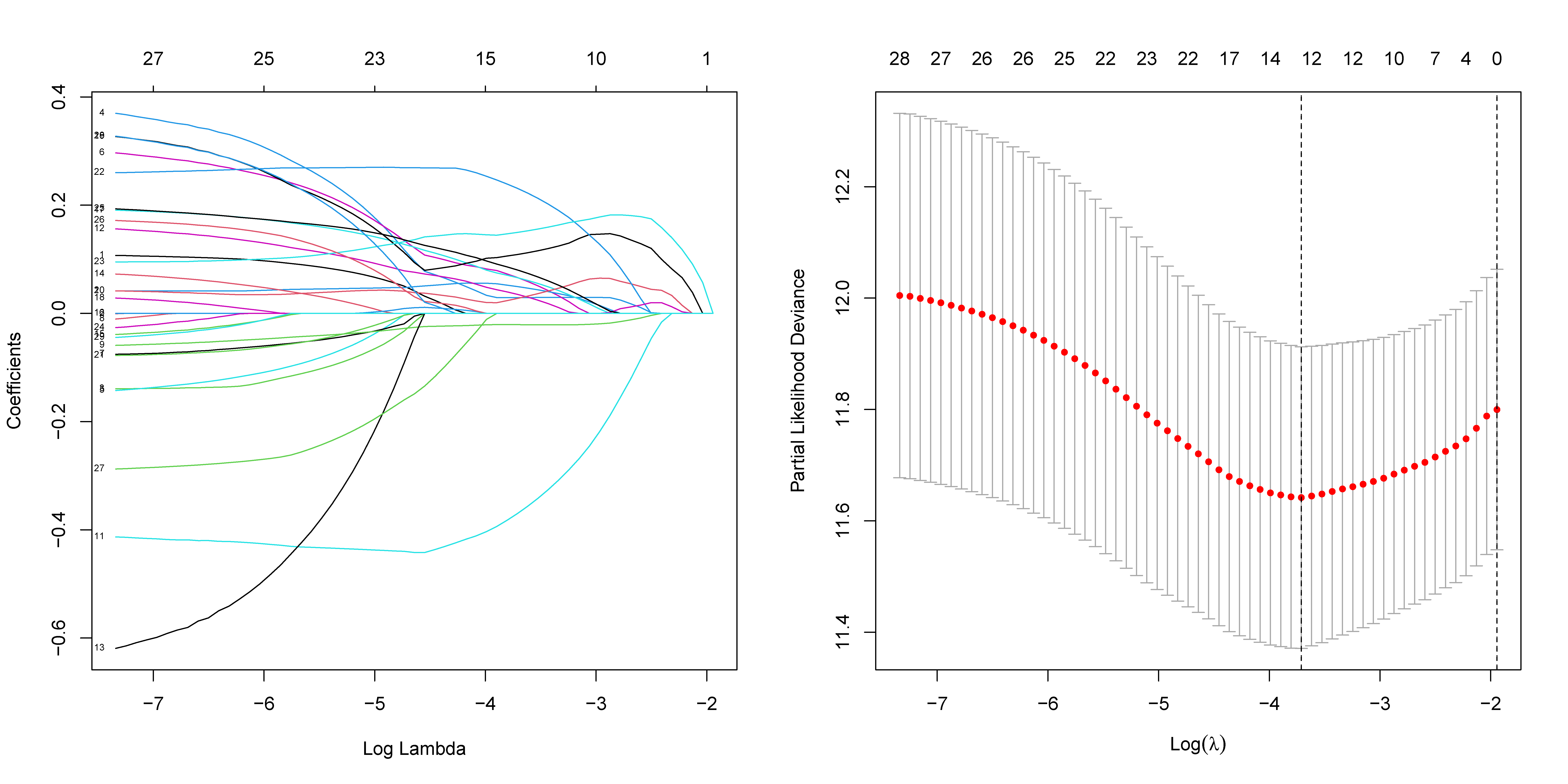

Supplement: Supplementary file 1 — Additional file 1: Figure S1. Lasso cox regression analysis was conducted to establish a prognostic signature including 12 platelet-related genes (optimum λ = 12). [file 12575_2022_185_MOESM1_ESM.tif]

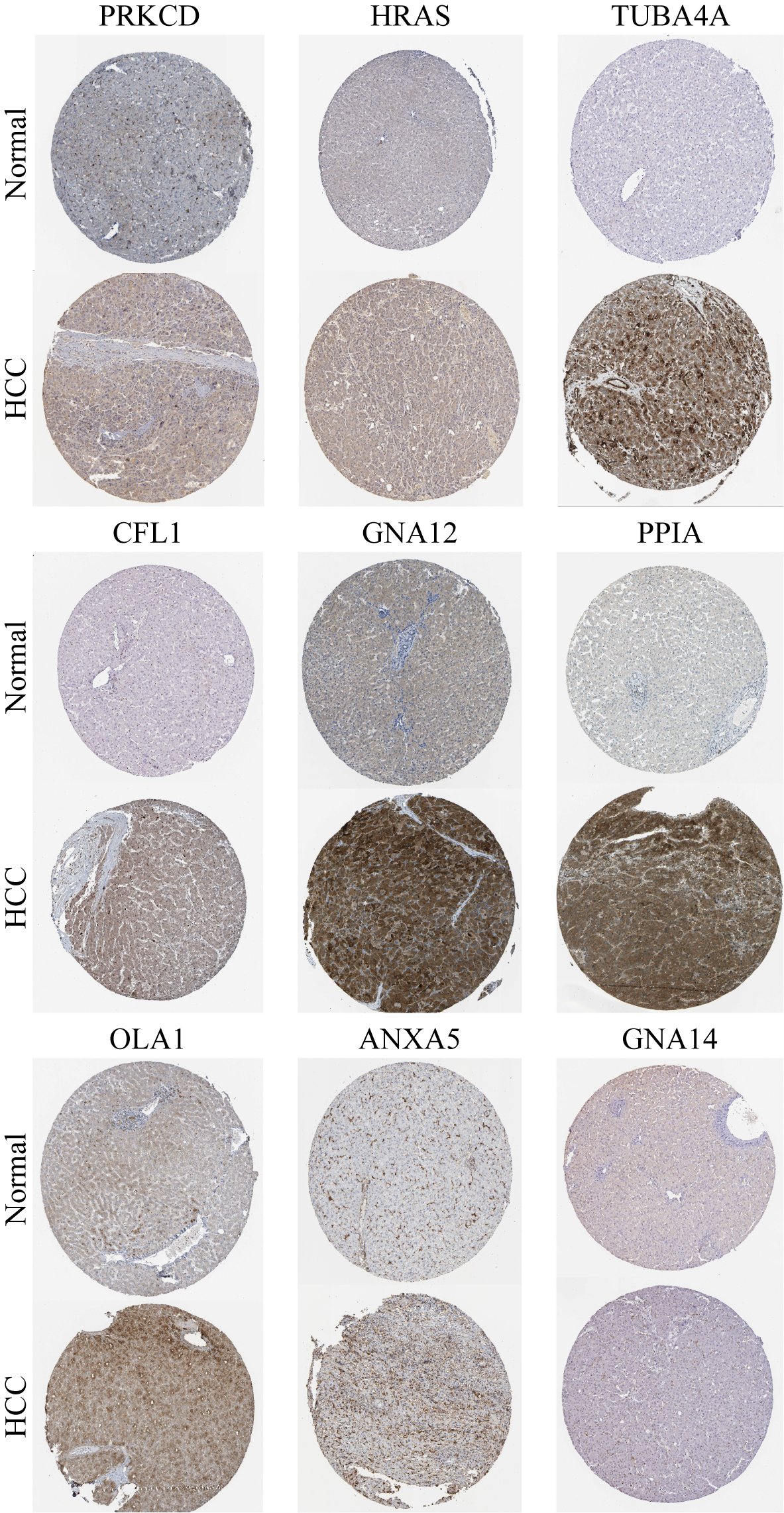

Supplement: Supplementary file 2 — Additional file 2: Figure S2. Immunohistochemistry (IHC) staining results of protein expression verification of PRKCD, HRAS, TUBA4A, CFL1, GNA12, PPIA, OLA1, ANXA5, and GNA14 between normal and HCC tissues in the HPA database. The results displayed that except for GNA14, the protein expression levels of other risk genes were significantly increased in tumor tissues, which was consistent with consistent with their expression patterns at the mRNA levels. [file 12575_2022_185_MOESM2_ESM.tif]

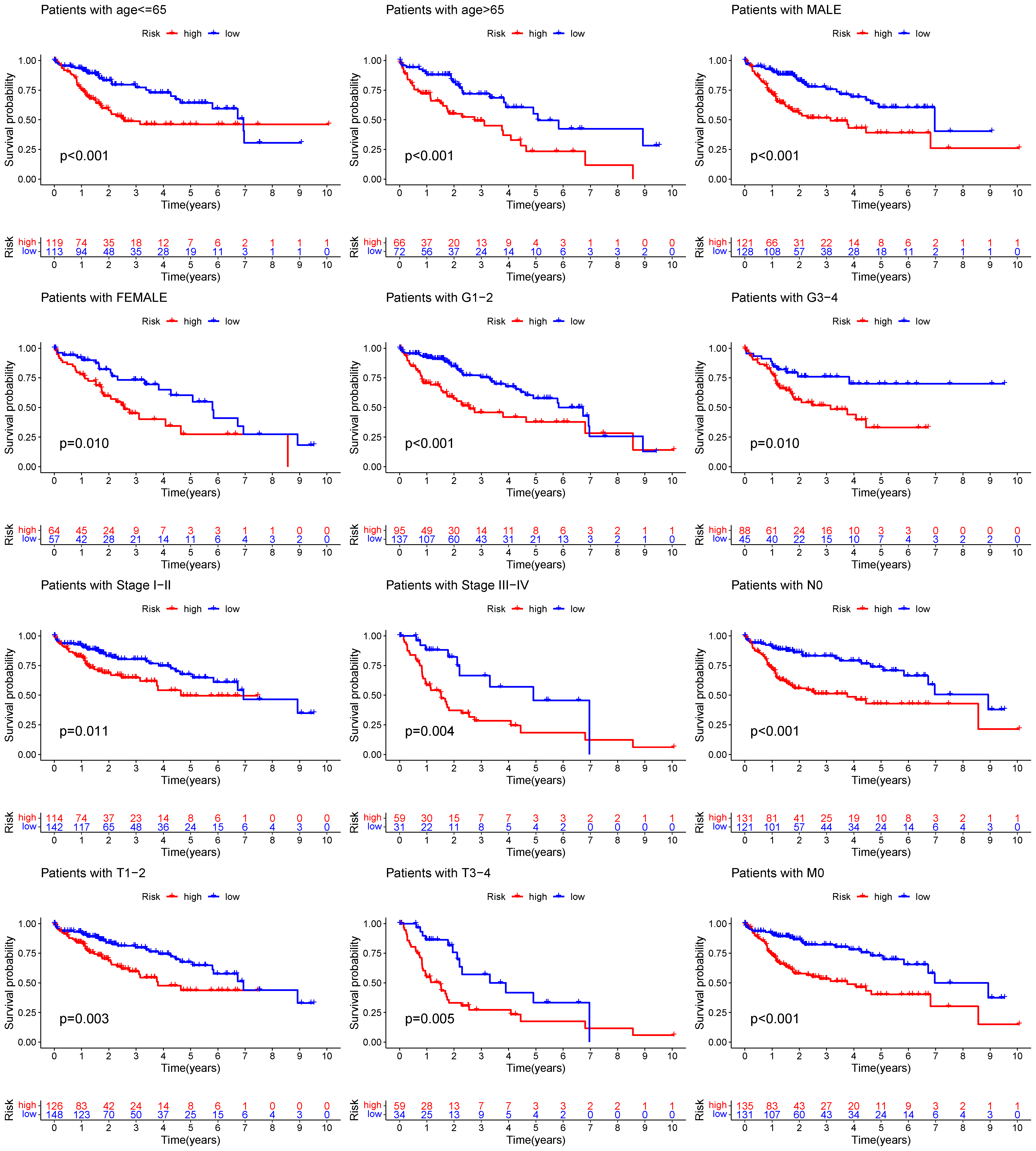

Supplement: Supplementary file 3 — Additional file 3: Figure S3. Kaplan-Meier survival curves of patients with different clinicopathological parameters (age, gender, pathological grade, tumor stage, and TNM stage) both in the high- and low-risk groups in the TCGA-LIHC cohort. [file 12575_2022_185_MOESM3_ESM.tif]

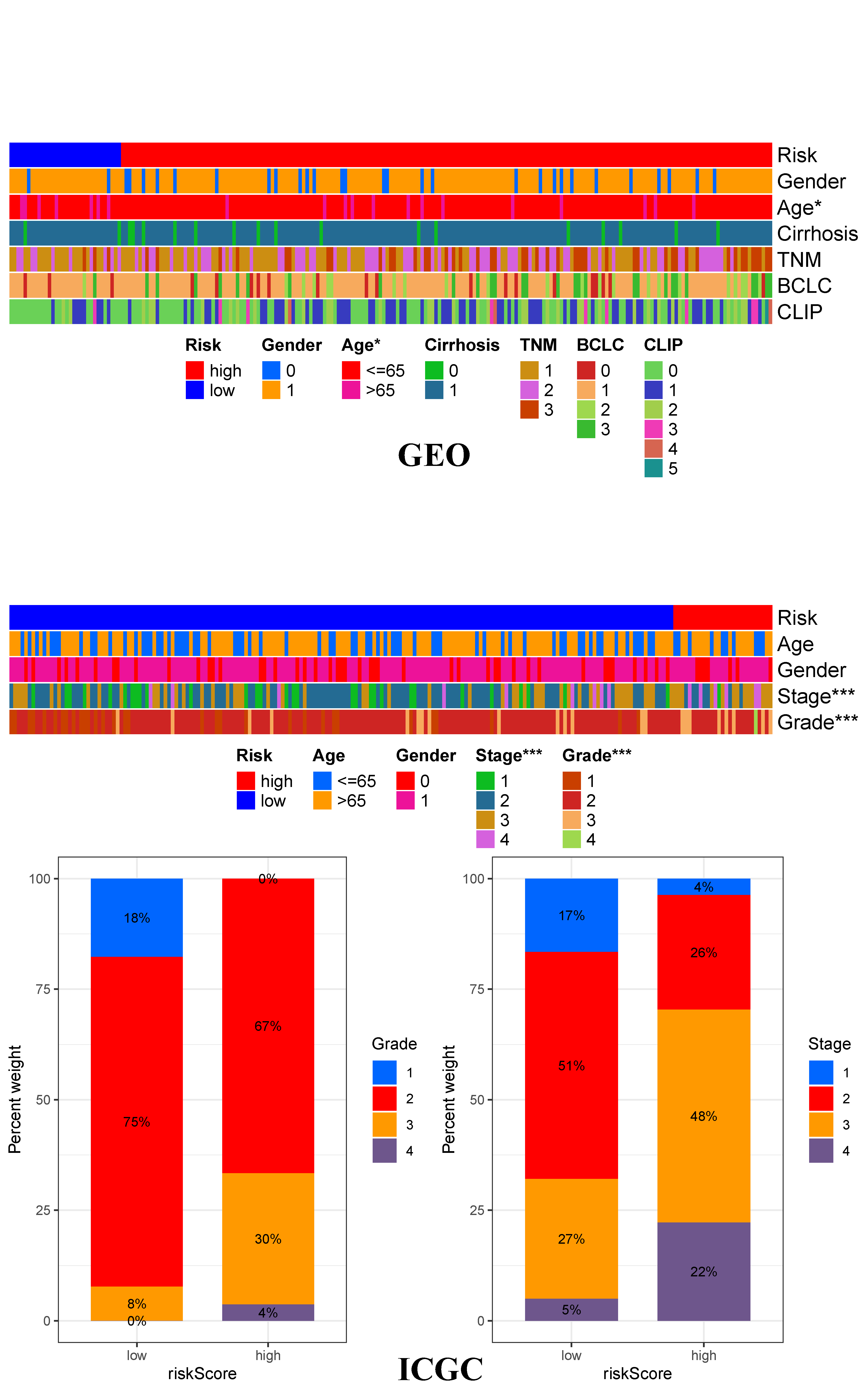

Supplement: Supplementary file 4 — Additional file 4: Figure S4. Clinical correlation analysis of HCC patients at different risk scores in the GSE14520 and ICGC-LIRI cohorts. A significant association between patients’ age and high-risk score was observed in the GSE14520 cohort, and an obviously correlation between pathological grade (G3), tumor stage (Stage III-IV), and high-risk score in the ICGC-LIRI cohort. (*p < 0.05; ***p < 0.001). [file 12575_2022_185_MOESM4_ESM.tif]

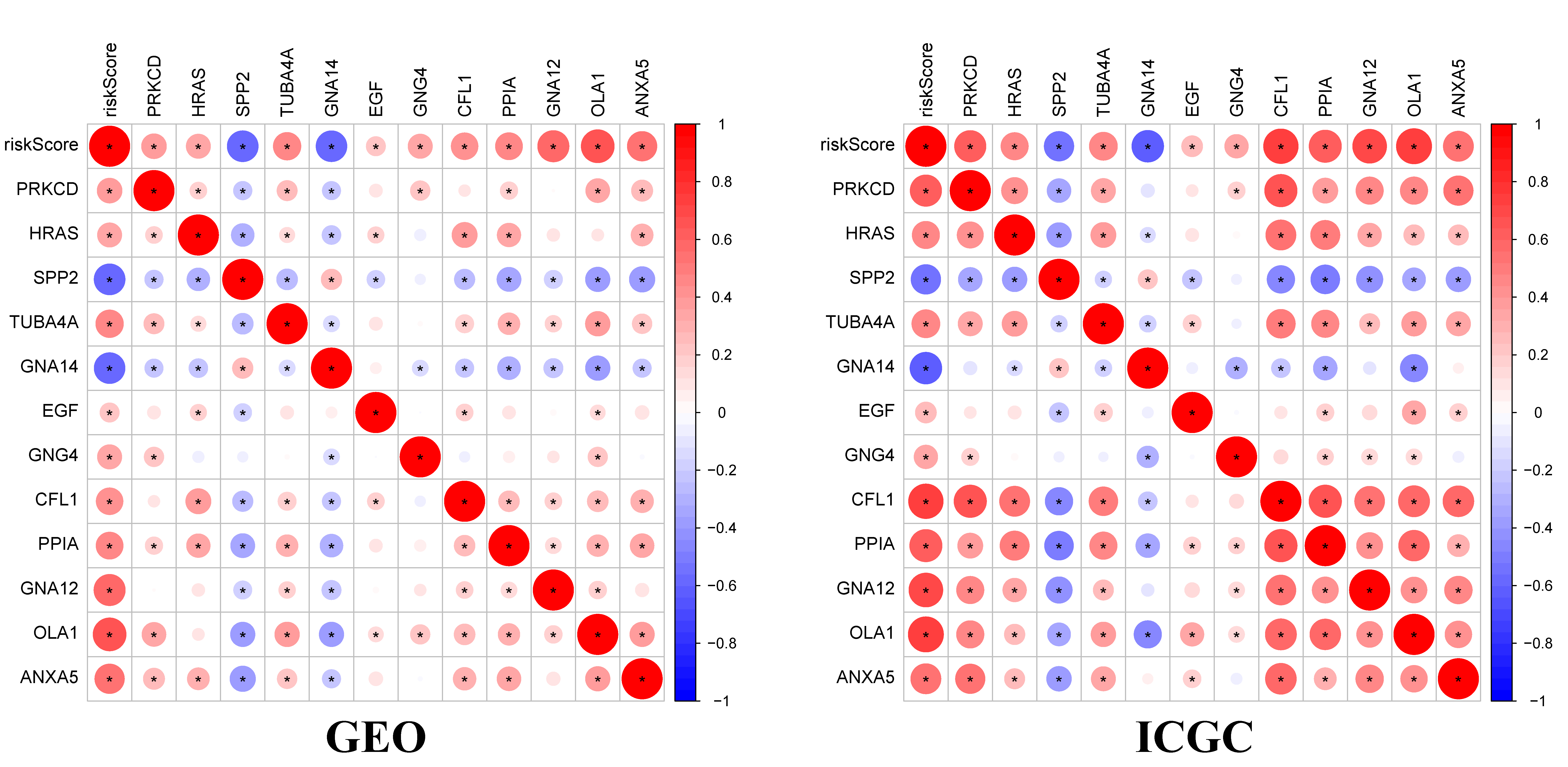

Supplement: Supplementary file 5 — Additional file 5: Figure S5. Association among risk genes in the GSE14520 and ICGC-LIRI cohorts analyzed by Spearman’s correlation test. (*p < 0.05). [file 12575_2022_185_MOESM5_ESM.tif]

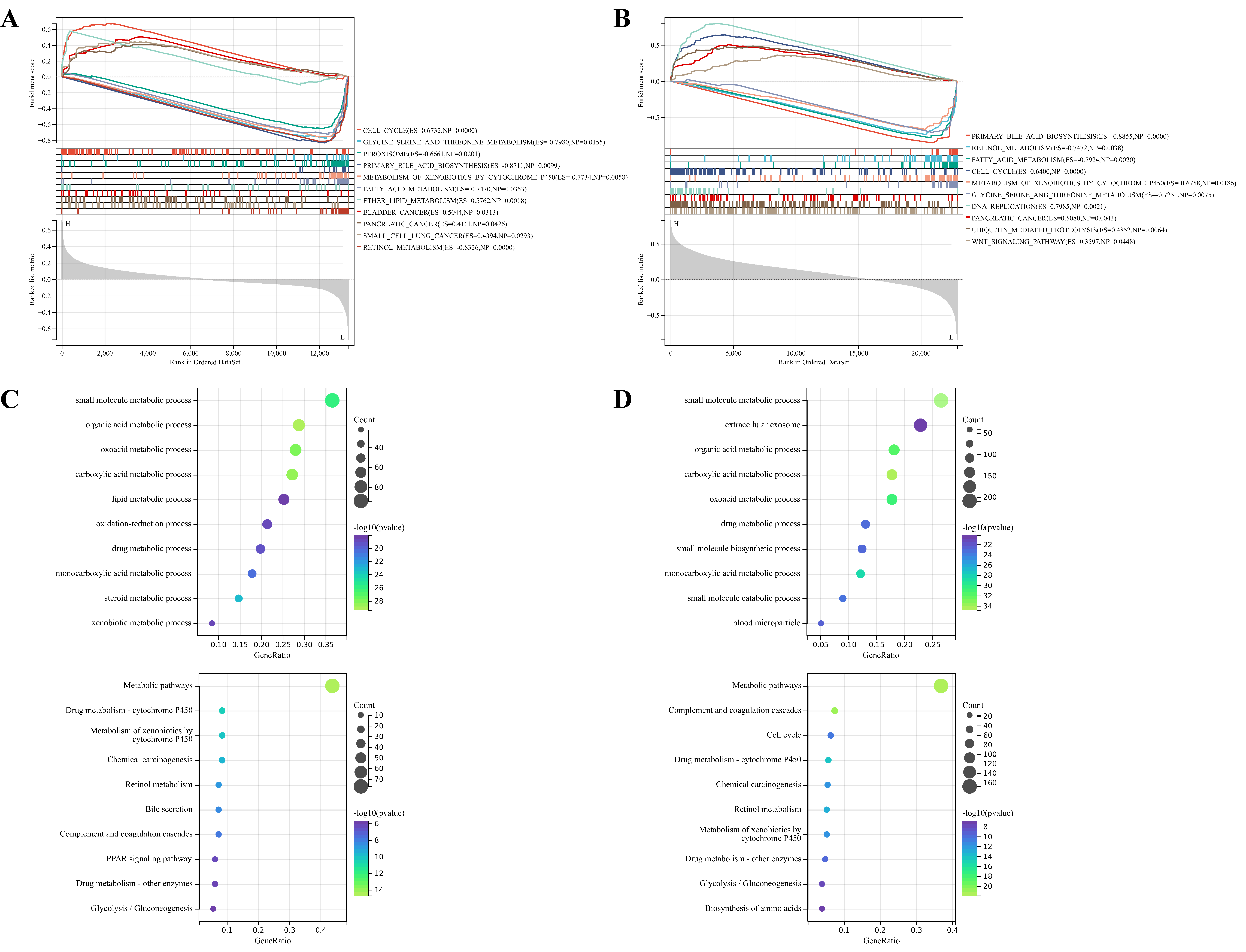

Supplement: Supplementary file 6 — Additional file 6: Figure S6. Enrichment analysis in the GSE14520 and ICGC-LIRI cohorts. (A) GSEA results with top 10 KEGG pathways (such as cell cycle and cancer-related pathways enriched in the high-risk group) in the GSE14520 cohort. (B) GSEA results with top 10 KEGG pathways (including cell cycle, DNA replication, ubiquitin mediated proteolysis, and wnt-signaling pathways enriched in the high-risk group) in the ICGC-LIRI cohort. (C) GO (upper) and KEGG (below) results based on the DEGs (|logFC| > 1 and p < 0.01) between different risk groups in the GSE14520 cohort. (D) GO (upper) and KEGG (below) results based on the DEGs (|logFC| > 1 and p < 0.01) between different risk groups in the ICGC-LIRI cohort. [file 12575_2022_185_MOESM6_ESM.tif]

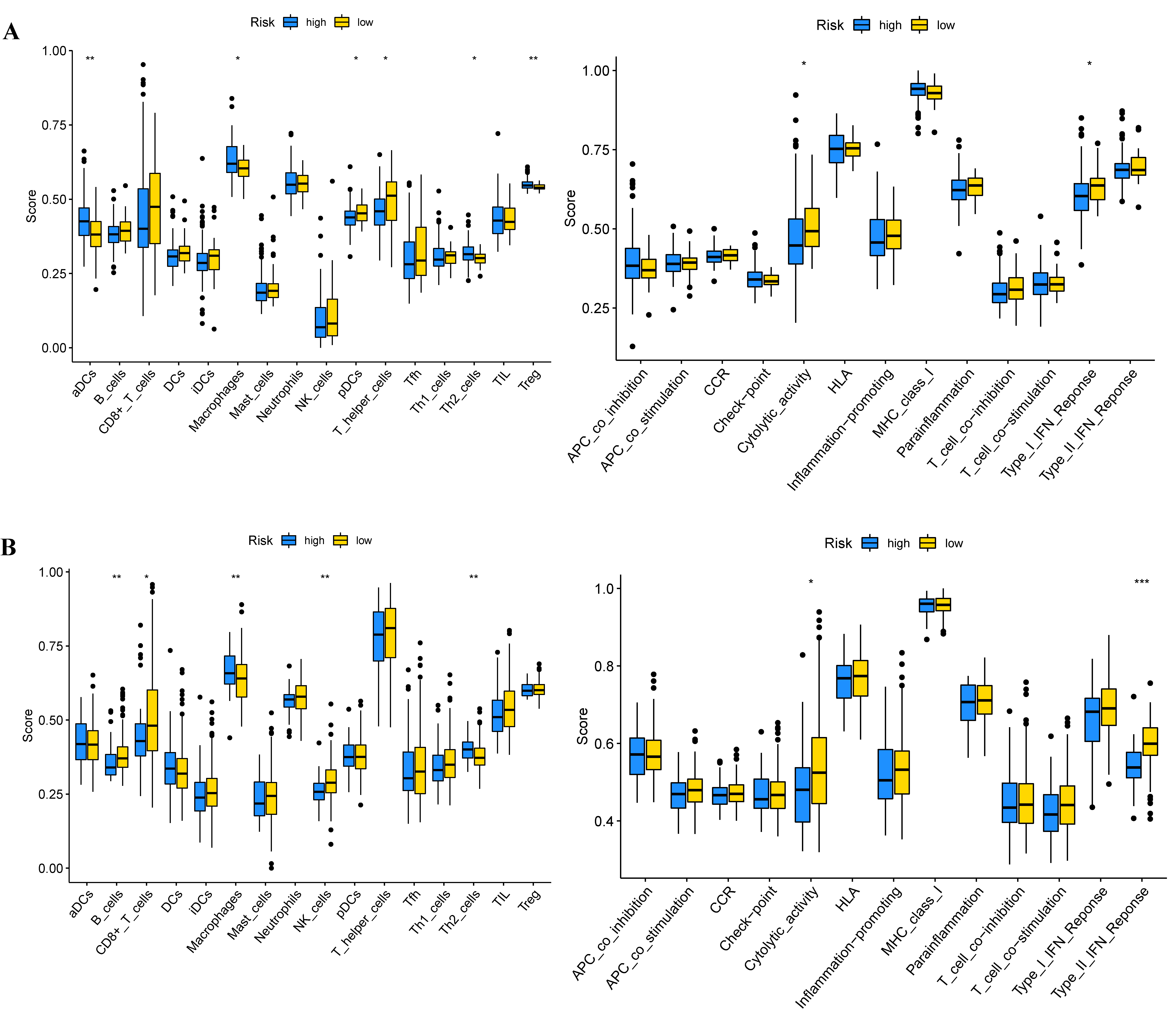

Supplement: Supplementary file 7 — Additional file 7: Figure S7. Score comparison of immune cells and functions between different risk groups in the GSE14520 (A) and ICGC-LIRI (B) cohorts. (*p < 0.05; **p < 0.01; ***p < 0.001). [file 12575_2022_185_MOESM7_ESM.tif]

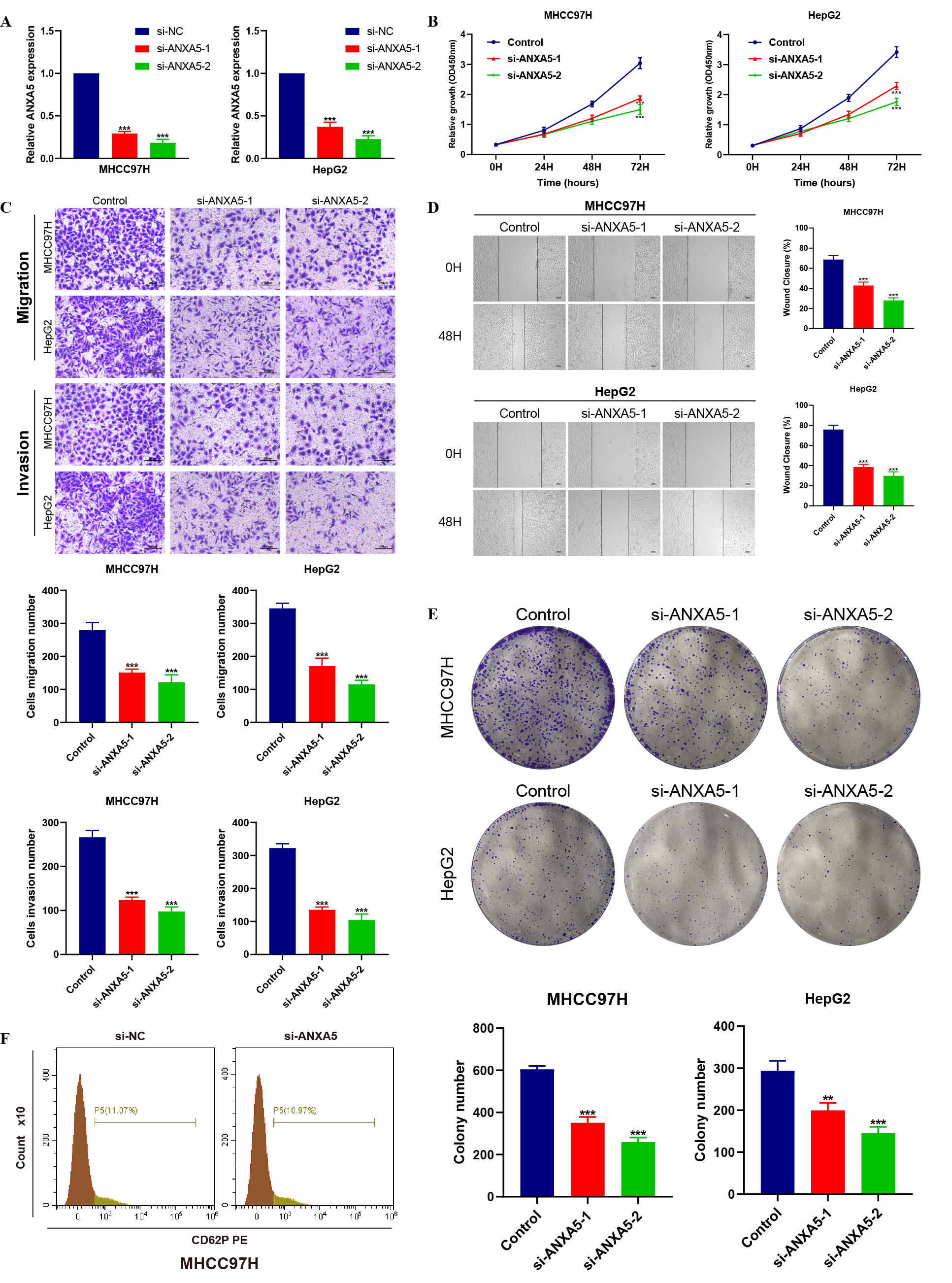

Supplement: Supplementary file 8 — Additional file 8: Figure S8. In vitro validation of ANXA5 on HCC cells proliferation, invasion and migration, as well as platelet activation. (A) Measurement of mRNA expression levels of ANXA5 in MHCC97H and HepG2 cells transfected with two ANXA5 siRNA sequences, si-NC was utilized as the negative control. (B) Effects of si-ANXA5 on proliferation abilities of MHCC97H and HepG2 cells measured by the CCK-8 assay. (C) The transwell assay was performed to assess the impacts of si-ANXA5 on HCC cells migration (upper) and invasion (below) capacities. Scale bar: 200 μm (200×). (D) The wound healing test displayed the migration ability of HCC cells undergone different treatments. Scale bar: 100 μm (40×). (E) Effects of si-ANXA5 on proliferation abilities of MHCC97H and HepG2 cells measured by the colony formation test. (F) Flow cytometry was performed to determine the effect of knockdown ANXA5 on platelet activation in MHCC97H cell and platelet co-culture system. The results were presented with representative images from three times independent replicate experiments, and all data were shown as Means ± SD. (**p < 0.01; ***p < 0.001). [file 12575_2022_185_MOESM8_ESM.tif]

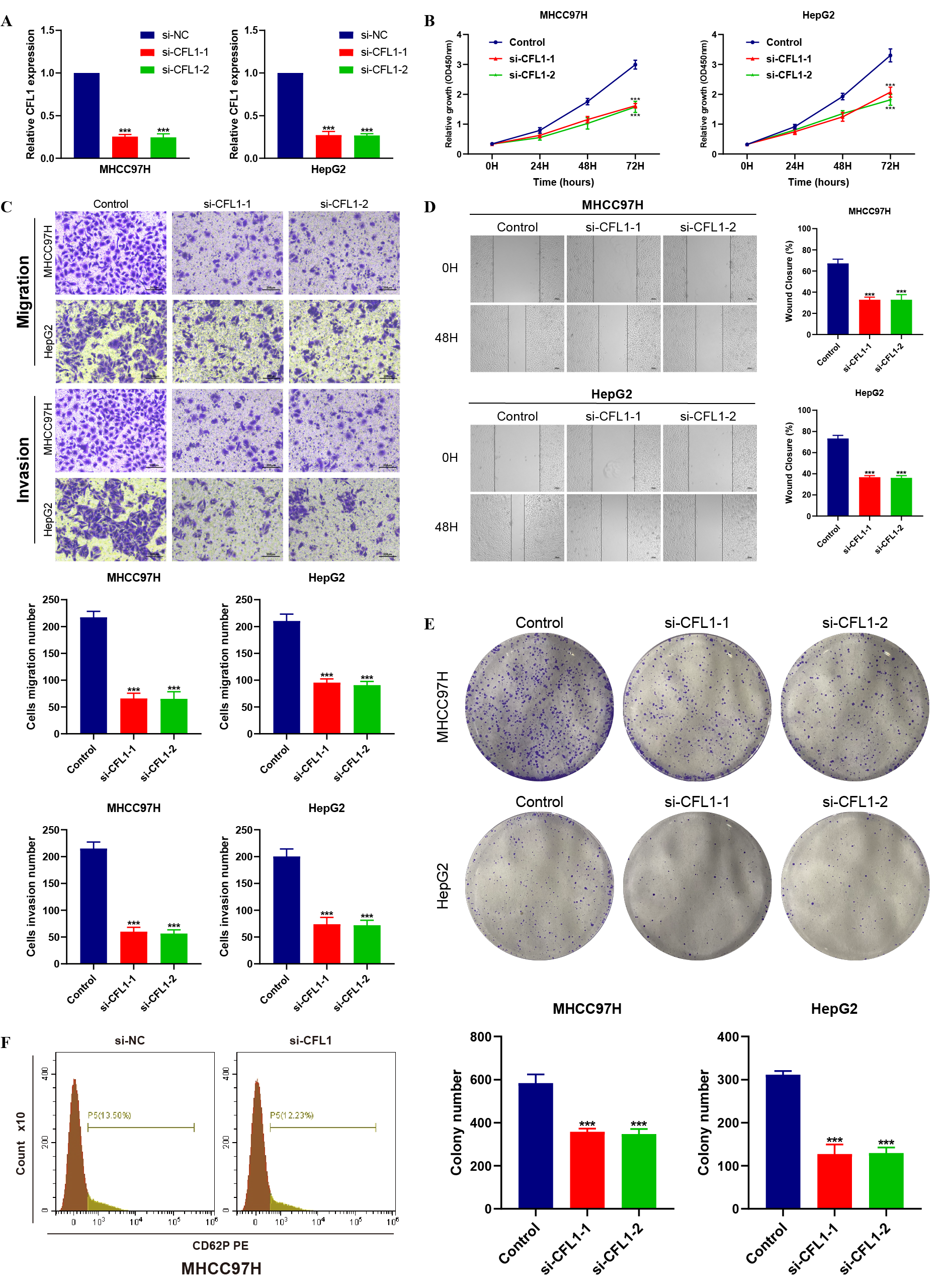

Supplement: Supplementary file 9 — Additional file 9: Figure S9. In vitro validation of CFL1 on HCC cells proliferation, invasion and migration, as well as platelet activation. (A) Measurement of mRNA expression levels of CFL1 in MHCC97H and HepG2 cells transfected with two CFL1 siRNA sequences, si-NC was utilized as the negative control. (B) Effects of si-CFL1 on proliferation abilities of MHCC97H and HepG2 cells measured by the CCK-8 assay. (C) The transwell assay was performed to assess the impacts of si-CFL1 on HCC cells migration (upper) and invasion (below) capacities. Scale bar: 200 μm (200×). (D) The wound healing test displayed the migration ability of HCC cells undergone different treatments. Scale bar: 100 μm (40×). (E) Effects of si-CFL1 on proliferation abilities of MHCC97H and HepG2 cells measured by the colony formation test. (F) Flow cytometry was performed to determine the effect of knockdown CFL1 on platelet activation in MHCC97H cell and platelet co-culture system. The results were presented with representative images from three times independent replicate experiments, and all data were shown as Means ± SD. (**p < 0.01; ***p < 0.001). [file 12575_2022_185_MOESM9_ESM.tif]
