# Supplementary material for: Genetic Analysis of Platelet-Related Genes in Hepatocellular Carcinoma Reveals a Novel Prognostic Signature and Determines PRKCD as the Potential Molecular Bridge
Source: Biol Proced Online. 2022 Dec 3;24:22. doi: 10.1186/s12575-022-00185-9 (PMC9719151; doi:10.1186/s12575-022-00185-9)
Supplement: Supplementary file 10 — Additional file 10: Table S1. 300 Platelet-related genes. Table S2. Primer sequences of genes in the risk signature. Table S3. 12 PRGs extracted via Lasso regression analysis. [file 12575_2022_185_MOESM10_ESM.docx]

**Table S1 300 Platelet-related genes**

| PDPN  SOS1  RAF1  HRG  PLA2G4A  PDGFB  PRKCQ  SELP  PLEK  SHC1  LYN  PRKCB  PIK3R1  MAPK1  F2  PIK3CG  F2RL3  PIK3CA  FCER1G  GRB2  SYK  PDGFA  CD9  F2R  MAPK3  PRKCA  PRKCD  TLR4  ADAMTS18  UBASH3B  ALOX12  ITGB1  RASA1  JAK1  ELK1  SERPINE2  PDGFRA  STAT3  PLCB1  TXK  TBXAS1  PRKG1  PLCG1  MAP2K1  DMTN  APOE  F11R  FOS  CEACAM1  PTGS1 | HRAS  MAP2K4  MAPK8  JUN  MAP3K1  CSNK2A1  TEC  SH2B3  BLK  SRF  STAT1  CELA2A  NOS3  STAT5A  C1QTNF1  THBD  ACTN4  TEX264  SCG3  PRKCE  GNAT3  DGKA  GNB5  PLG  VCL  ADRA2C  GNG8  LCP2  CDC37L1  SERPINE1  ARRB2  GP6  MPL  PCYOX1L  FAM3C  MANF  IGF1  MAPK14  TGFB2  RAC1  SPP2  GP1BA  SYTL4  TUBA4A  P2RY1  GNA14  CSK  RAB27B  TLN1  RAP1A | PRKCG  LCK  TTN  STXBP2  PECAM1  PTPN1  EGF  GNAI3  F2RL2  DGKZ  PRKCZ  FYN  DAGLA  LAMP2  DGKE  VEGFC  LEFTY2  F13A1  MGLL  CHID1  CRK  BRPF3  PLCG2  LGALS3BP  ITGA2B  F5  PSAP  GNAI2  LHFPL2  TGFB3  DGKG  THPO  QSOX1  AKT1  PRKCH  TOR4A  A2M  PIK3R5  PDPK1  TIMP3  IGF2  ISLR  RARRES2  VEGFD  RAC2  COL1A1  NHLRC2  GP1BB  RAP1B  GNG3 | DGKI  RASGRP1  PTPN11  ORM2  ADRA2B  ITPR1  AHSG  CAP1  AAMP  DGKH  DAGLB  TAGLN2  BCAR1  CLEC1B  GNA15  GNG4  GNAI1  PFN1  DGKB  P2RY12  ITGB3  GP9  CDC42  VWF  SOD1  GP5  GNB1  TMX3  PF4  CYB5R1  WDR1  CYRIB  GNG13  APOH  ARRB1  FGA  MMRN1  ORM1  SCCPDH  SPARC  CALU  PTK2  ITIH3  LAT  DGKQ  FGG  HSPA5  ALB  CFL1  APOOL | SERPING1  RHOB  GNAQ  PCDH7  VTI1B  SRGN  PPIA  GNG5  ACTN1  APOA1  CD36  CLU  GNG2  PIK3CB  ENDOD1  GNGT2  PIK3R6  PROS1  PHACTR2  VEGFA  TF  GNG10  CTSW  ADRA2A  CD63  GNA12  TGFB1  OLA1  VAV2  TMSB4X  MAGED2  ITPR2  A1BG  GAS6  SERPINA3  THBS1  GNA11  RAPGEF3  PIK3R2  APBB1IP  SERPINF2  RASGRP2  GNB2  LY6G6F  PPBP  TIMP1  APLP2  CALM1  FGB  ABHD12 | FERMT3  ALDOA  ABHD6  GNG12  FLNA  HABP4  F8  TRPC6  COL1A2  GNA13  GNGT1  VAV3  RAPGEF4  TBXA2R  CLEC3B  STXBP3  RHOA  ITIH4  ACTN2  TRPC7  HGF  SELENOP  SERPINA1  CFD  TRPC3  STX4  SERPINA4  GNB4  VAV1  ANXA5  APP  PTPN6  MPIG6B  GTPBP2  ECM1  VEGFB  CD109  RHOG  SRC  GNG11  KNG1  FN1  ABCC4  GNG7  GNB3  DGKD  ITPR3  PIK3R3  DGKK  YWHAZ |
| --- | --- | --- | --- | --- | --- |

| **Gene** | **Forward Primer** | **Reverse Primer** |
| --- | --- | --- |
| PRKCD | GTGCAGAAGAAGCCGACCAT | CCCGCATTAGCACAATCTGGA |
| HRAS | ATGACGGAATATAAGCTGGTGGT | GGCACGTCTCCCCATCAATG |
| SPP2 | TTGGAATGAACTACTGGTCTTGC | CCCGAAACAGATACGGACTCAG |
| TUBA4A | TGAGATCCGAAATGGCCCATA | TAGTGACCACGGGCATAGTTG |
| GNG4 | GAGGGCATGTCTAATAACAGCAC | AGACCTTGACCCTGTCCATAC |
| GNA12 | CCGCGAGTTCGACCAGAAG | TGATGCCAGAATCCCTCCAGA |
| GNA14 | GAGCGATGGACACGCTAAGG | TCCTGTCGTAACACTCCTGGA |
| PPIA | CCCACCGTGTTCTTCGACATT | GGACCCGTATGCTTTAGGATGA |
| EGF | TGGATGTGCTTGATAAGCGG | ACCATGTCCTTTCCAGTGTGT |
| CFL1 | TTCAACGACATGAAGGTGCGT | TCCTCCAGGATGATGTTCTTCT |
| OLA1 | TTGCAGCACTCCAACTAGAATAC | TCGGTTGTTGAGGTGTGTTAAAT |
| ANXA5 | AGCGGGCTGATGCAGAAAC | ACTTCGGGATGTCAACAGAGT |
| ACTB  E-cadherin  N-cadherin  Vimentin  Snail | GGACTTCGAGCAAGAGATGG  CGAGAGCTACACGTTCACGG  TCAGGCGTCTGTAGAGGCTT  GACGCCATCAACACCGAGTT  TCGGAAGCCTAACTACAGCGA | AGCACTGTGTTGGCGTACAG  GGGTGTCGAGGGAAAAATAGG  ATGCACATCCTTCGATAAGACTG  CTTTGTCGTTGGTTAGCTGGT  AGATGAGCATTGGCAGCGAG |

**Table S2 Primer sequences of genes in the risk signature.**

**Table S3 12 PRGs extracted via Lasso regression analysis**

| Lasso PRGs | Coefficient |
| --- | --- |
| PRKCD  HRAS  SPP2  TUBA4A  GNA14  EGF  GNG4  CFL1  PPIA  GNA12  OLA1  ANXA5 | 0.029  0.066  -0.021  0.050  -0.370  0.035  0.066  0.109  0.028  0.231  0.150  0.080 |
